# Strategies for intrapartum foetal surveillance in low- and middle-income countries: A systematic review

**DOI:** 10.1371/journal.pone.0206295

**Published:** 2018-10-26

**Authors:** Natasha Housseine, Marieke C. Punt, Joyce L. Browne, Tarek Meguid, Kerstin Klipstein-Grobusch, Barbara E. Kwast, Arie Franx, Diederick E. Grobbee, Marcus J. Rijken

**Affiliations:** 1 Department of Obstetrics and Gynaecology, University Medical Centre Utrecht, Utrecht University, Utrecht, The Netherlands; 2 Julius Global Health, Julius Centre for Health Sciences and Primary Care, University Medical Centre Utrecht, Utrecht University, Utrecht, The Netherlands; 3 Department of Obstetrics and Gynaecology, Mnazi Mmoja Hospital, Zanzibar, Tanzania; 4 School of Health and Medical Science, State University of Zanzibar (SUZA), Zanzibar, Tanzania; 5 Division of Epidemiology & Biostatistics, School of Public Health, Faculty of Health Sciences, University of the Witwatersrand, Johannesburg, South Africa; 6 International Consultant Maternal Health and Safe Motherhood, Leusden, The Netherlands; University of Heidelberg, GERMANY

## Abstract

**Background:**

The majority of the five million perinatal deaths worldwide take place in low-resource settings. In contrast to high-resource settings, almost 50% of stillbirths occur intrapartum. The aim of this study was to synthesise available evidence of strategies for foetal surveillance in low-resource settings and associated neonatal and maternal outcomes, including barriers to their implementation.

**Methods and findings:**

The review was registered with Prospero (CRD42016038679). Five databases were searched up to May 1^st,^ 2016 for studies related to intrapartum foetal monitoring strategies and neonatal outcomes in low-resource settings.

Two authors extracted data and assessed the risk of bias for each study. The outcomes were narratively synthesised. Strengths, weaknesses, opportunities and threats analysis (SWOT) was conducted for each monitoring technique to analyse their implementation.

There were 37 studies included: five intervention and 32 observational studies. Use of the partograph improved perinatal outcomes. Intermittent auscultation with Pinard was associated with lowest rates of caesarean sections (10–15%) but with comparable perinatal outcomes to hand-held Doppler and Cardiotocography (CTG). CTG was associated with the highest rates of caesarean sections (28–34%) without proven benefits for perinatal outcome. Several tests on admission (admission tests) and adjunctive tests including foetal stimulation tests improved the accuracy of foetal heart rate monitoring in predicting adverse perinatal outcomes.

**Conclusions:**

From the available evidence, the partograph is associated with improved perinatal outcomes and is recommended for use with intermittent auscultation for intrapartum monitoring in low resource settings. CTG is associated with higher caesarean section rates without proven benefits for perinatal outcomes, and should not be recommended in low-resource settings. High-quality evidence considering implementation barriers and enablers is needed to determine the optimal foetal monitoring strategy in low-resource settings.

## Introduction

Over two million stillbirths are estimated to occur yearly worldwide, of which >98% are in low-resource settings [1,2]. Almost half of the number of stillbirths in low- and middle-income countries (LMICs) occur during labour, whereas most stillbirths in high-income countries (HICs) take place during the antenatal period [3,4]. The time of labour and delivery is a challenging period for the foetus and can result in foetal asphyxia and associated irreversible organ damage and mortality [5–8]. Intrapartum foetal monitoring allows for prompt and effective intervention when needed, and avoids unnecessary interventions like caesarean sections (CS) by offering confirmation of a favourable foetal condition [9]. Methods of foetal surveillance include foetal heart rate (FHR) monitoring by intermittent auscultation (IA), cardiotocography (CTG) with foetal blood sampling and foetal electrocardiogram with ST-wave analysis [10,11]. Nearly all methods are considered to be high-tech, complex in operation, and require significant financial resources [12,13].

Although global consensus exists that some form of foetal monitoring should be used during labour to improve maternal and neonatal outcomes, there is no evidence for an ideal foetal monitoring system [8,11,14]. Studies on foetal monitoring have been primarily conducted in HICs and, based on variable level of evidence, consensus-based guidelines were developed for foetal surveillance, which may not be readily applicable to LMICs due to context-specific factors [11,15–17]. Thus, in many low resource settings, low-cost and low-tech methods such as IA by Pinard stethoscope or hand-held Doppler, are the only accessible methods [18]. A review on intrapartum foetal surveillance (implementation) strategies for LMICs is not available. Therefore, the aim of this systematic review was to synthesize the available evidence for intrapartum foetal surveillance in low resource settings and a SWOT analysis was applied to analyse the implementation.

## Methods

This review was registered with the PROSPERO registry for systematic reviews (CRD42016038679). It adhered to PRISMA guidelines (S1 File) [19] and was conducted according to the Cochrane methodology [20].

### Research questions

This review aimed to answer two research questions: (1) what is the available evidence for strategies of intrapartum foetal surveillance in low- and middle-income countries and their associated neonatal and maternal outcomes? (2) what are the strengths, weaknesses, opportunities, and threats (SWOT) associated with the implementation of these intrapartum foetal surveillance strategies?

### Eligibility criteria

Observational or intervention studies concerning women receiving intrapartum foetal surveillance with reported neonatal outcomes in low resource settings were eligible for inclusion. These included studies on admission tests, which were defined as tests performed to determine foetal wellbeing upon arrival in labour in a birth facility. Low resource settings were defined as low-income, lower-middle- and upper-middle income countries (LICs, L-MICs, and UMICs respectively), according to the World Bank classification [21]. Conference abstracts, reports, editorials, presentations, and project protocols were excluded.

### Information sources and search

The search was conducted in the following electronic databases: Pubmed/MEDLINE, The Cochrane Library, EMBASE, POPLINE and Global Health Library to include all articles up to May 1^st^, 2016. For every database, a search string was developed with the support of a librarian specialised in medical sciences, using pre-defined search (Title/Abstract) and MeSH/Emtree terms when applicable. References were manually searched for additional studies. Only for the Global Health Library, limits were used (humans/English). The full search strings are available in Appendix A in S2 File.

### Study selection

Mendeley reference software was used to remove duplicates. Subsequently, two reviewers (MCP and NH) independently screened articles based on title and abstract, after which full-text screening was performed. In case of disagreement, a third reviewer (MJR) was consulted. Authors were contacted once in case of inaccessible full-texts, and a study excluded if no reply was received.

### Data collection process

Data extraction of the included studies was conducted by one reviewer (MCP) and double-checked for accuracy by a second reviewer (NH). A standardised data extraction sheet was created (Appendix B in S2 File) SWOT analysis was applied to the methods, results and discussion sections of the selected articles whenever mentioned and recorded in the same extraction sheet as all other outcomes. Outcome measurements were noted as percentages and calculated when possible in case of different reporting strategy. Sensitivity, specificity, positive and negative predictive values (PPV and NPV respectively) were collected when available. The corresponding author or organisation was emailed once in case of incomplete data. In case of disagreements during the extraction process, other members of the review team were contacted (JB, MJR).

### Risk of bias assessment

The level of bias was assessed for each study using the Cochrane Risk of Bias Tool (S3 File) and the Newcastle-Ottawa Quality Assessment Scale for intervention and observational studies, respectively (S4 and S5 File) [20,22,23]. Colour coding of the table was assigned as red, green and yellow for high, low and unclear (Cochrane) or intermediate (Newcastle-Ottawa) risk respectively. Judgement of bias was determined (MCP) and double-checked for accuracy (NH). Any disagreement during this process was resolved by contacting other members of the review team (JB, MJR).

### Data synthesis

Due to heterogeneity in domains, determinants, study designs and reported outcomes, a senior statistician from the Cochrane Collaboration advised not to conduct a meta-analysis. This review, therefore, consists of a narrative analysis of strategies for intrapartum foetal surveillance and their corresponding outcomes. The quantitative results of all studies were summarised according to study design: intervention and descriptive studies. For each method of foetal monitoring, SWOT findings were summarised according to each component.

## Results

**Fig 1:**
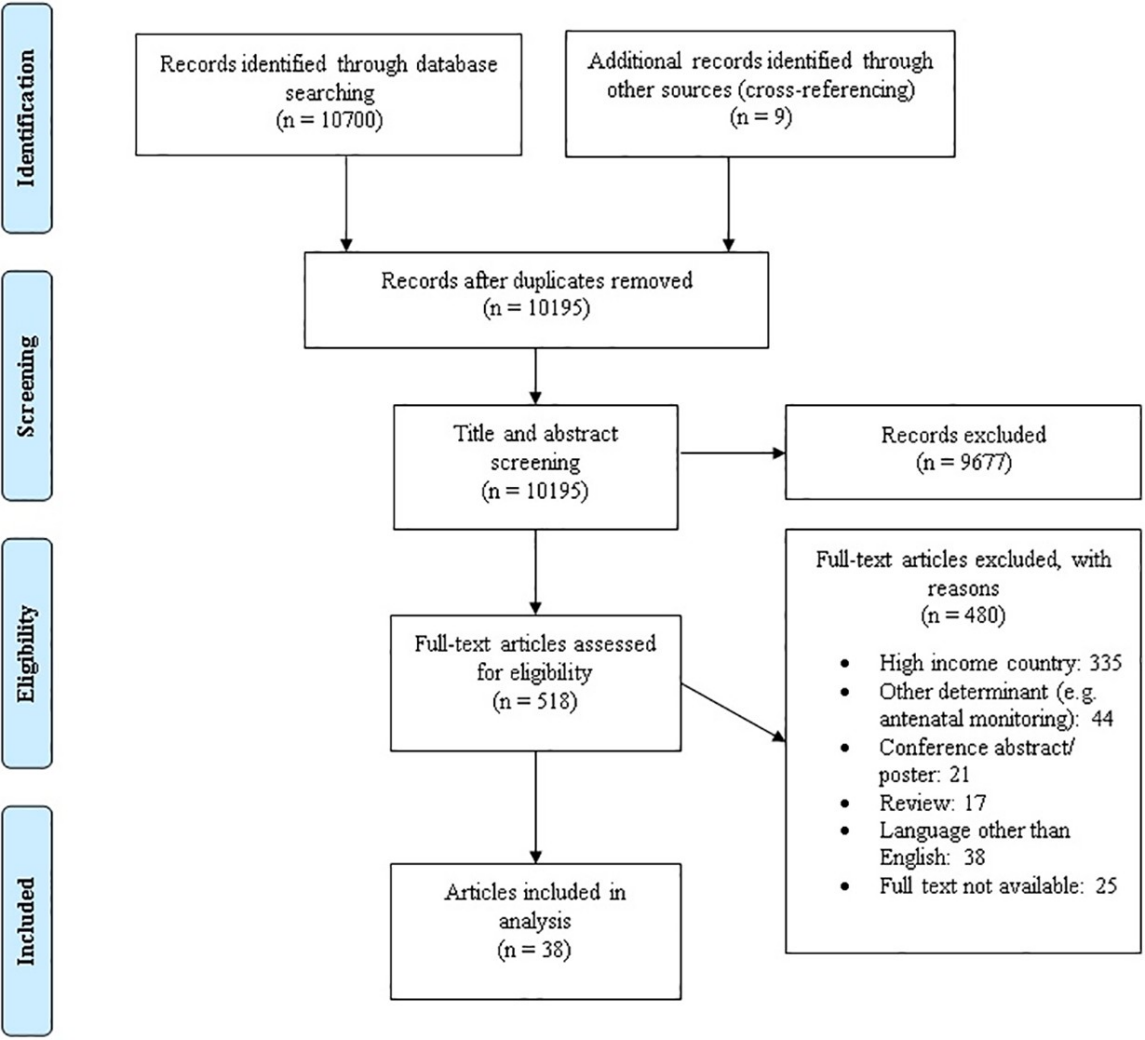
Flow diagram of search results.

A total of 10,195 articles were obtained after removal of duplicates and including nine articles from cross-referencing (Fig 1). After title- and abstract screening, 518 articles were screened in full-text, of which 38 were included. Two publications reported on the same study [24,25]. The final 37 included studies consisted of five (13.5%) intervention studies (three randomised controlled trials (RCT)[26–28] and two clustered RCT [24,25,29]), and 32 (86.5%) observational studies (23 cohort studies, six cross-sectional studies, and three case-control studies) [30–60]. The studies were conducted in Africa (n = 16), Asia (n = 21) and Europe (n = 1) and were from LICs (n = 6), L-MICs (n = 21) and UMICs (n = 11). Many studies were from urban settings (urban: n = 16, rural: n = 1, both: 2), for 18 studies this could not be determined. Studies were on: admission tests and early intrapartum (CTG, n = 7, IA: 1, other methods, n = 6) [32–35,44,48,51,56,57,59,61], ongoing intrapartum FHR monitoring (IA, n = 8; CTG, n = 11) [26–28,30,31,36–39,41,43,45–47,50,53,58], adjunctive tests (n = 9) [39,41–43,45,46,49,52,58] and partograph (n = 5) [24,25,29,40,54,55] (Tables 1–3).

**Table 1:** Quality assessment of randomised controlled trials (n = 5).

Randomised controlled trial	Intervention	Population characteristics	Sequence generation	Allocation concealment	Blinding of participant/ researcher	Selection of study population	Completeness of data	Origin of data	Clear definition of outcome?	Confounders taken into account?
Byaruhanga et al. 2015, Uganda [27]	Wind-up, Doppler vs Pinard	1971, singleton, cephalic, >37 weeks, mixed-risk	Unclear risk	Unclear risk	High risk	Low risk	Low risk	Low risk	Low risk	High risk
Fahdhy et al. 2005 Indonesia [29]*	WHO partograph and training	625 low risk	Low risk	Low risk	High risk	High risk	Low risk	Low risk	Low risk	Unclear risk
Madaan et al. 2006 India [28]	IA vs Continuous CTG	100 post caesarean section singleton	Unclear risk	Unclear risk	High risk	Low risk	Low risk	Low risk	Low risk	Unclear risk
Mahomed et al. 1994, Zimbabwe [26]	Intermittent CTG, Doppler, Pinard	1255 singleton, cephalic, >37weeks, mixed-risk	Unclear risk	Low risk	High risk	Low risk	Low risk	Low risk	Low risk	Low risk
WHO, 1994 & Lennox 1998 Southeast Asia [24,25]*	WHO Partograph	35 484, mixed-risk	Unclear risk	Unclear risk	High risk	Low risk	Low risk	Low risk	Low risk	Unclear risk

Colour coding: Green = Low risk, Red = High risk and Yellow = Unclear risk. Abbreviations: CTG = Cardiotocography, IA = Intermittent Auscultation

*Clustered randomised control trial

**Table 2:** Quality assessment of the observational studies (n = 32).

Cohort studies	Method/ strategy	Population character-istics*	Selection process	Compar-ability	Exposure	Cross-sectional studies	Method/ strategy	Population character-istics*	Selection process	Compar-ability	Outcome
Aboulghar et al. 2013, Egypt [30]	CTG	High risk	4	0	2	Adanikin et al. 2016 Nigeria [31]	IA	Mixed-risk	4	2	2
Bakr et al. 2005 Egypt [42]	FPO vs FBS	Unclear	4	0	2	Bolbol-Haghighi et al. 2015, Iran [55]	Partograph	Low risk	4	0	2
Chittacharoen et al. 2000, Thailand [56]	FAST and Admission CTG	High risk	4	0	3	Ogwang et al. 2009, Uganda [40]	Partograph	Unclear,	4	0	2
Chittacharoen et al. 1997, Thailand [61]	FAST	Unclear	4	0	3	Oladapo et al. 2009, Nigeria [41]	IA and MSAF	Mixed-risk	4	2	2
David et al. 2014 India [57]	Admission CTG	Low risk	4	0	2	Parveen et al. 2010, Pakistan [43]	CTG and MSAF	Low risk	5	0	1
Duhan et al. 2010 India [58]	MSAF and CTG	Unclear	3	1	2	Tasnim et al. 2009, Pakistan [50]	CTG	Mixed-risk	4	0	2
Goldenberg et al. 2013, Multi-country [59]	Admission IA(Doppler)	Unclear	4	0	2	**Case-control**	**Method/ strategy**	Population Character-istics*	**Selection process**	**Compar-ability**	**Exposure**
Goonewardene et al. 2011, Sri Lanka [60]	FAST and Admission CTG	Low risk	4	0	2	Bogdanovic et al. 2014, Bosnia [53]	CTG	Unclear	2	0	3
Gupta et al. 1997 India, [52]	MSAF	Mixed-risk	3	0	2						
Howarth et al. 1992, South Africa [33]	UADV	Unclear	4	0	3						
Kulkarni et al. 1998 India [34]	Admission CTG	High risk	4	0	2						
Kushtagi et al. 2011, India [35]	Admission AFI	Mixed-risk	4	0	3						
Langli Ersdal et al. 2012, Tanzania [36]	IA	Mixed-risk	4	2	2						
Odendaal et al. 1977, South Africa [37]	CTG	unclear	4	2	2						
Odendaal et al. 1994, South Africa [38]	CTG	High risk	4	2	2						
Odongo et al. 2010 Kenya [39]	CTG and MSAF	Unclear	3	1	2						
Rahman et al. 2012 India [32]	Admission CTG	Mixed risk	4	2	2						
Rathore et al. 2011 India [45]	FSST, IA and MSAF	High risk	4	2	2						
Raouf et al. 2015 Iran[44]	CTG	Low risk	3	0	2						
Rotich et al. 2006 Kenya [46]	IA and MSAF	Mixed-risk	3	2	3						
Roy et al. 2008 India [47]	CTG	Unclear	4	0	3						
Shaktivardhan et al. 2009, India [48]	Admission CTG	High risk	4	0	2						
Stuart et al. 1993 South Africa [49]	UADV	High risk	4	0							
Tongprasert et al. 2006 Thailand [51]	rBPP	Mixed-risk	4	0	3						

Colour coding: Green = Low -, Red = High—and Yellow = Intermediate risk of bias. Maximum points to be allocated (Cohort/ cross-sectional/ case-control): Selection process (4/5/4), Comparability (2/2/2), Outcome (3/3/-), Exposure (-/-/3). AFI = Amniotic fluid index, CTG = cardiotocograph, FAST = Foetal acoustic stimulation test, FBS = Foetal blood sampling, FPO = Foetal pulse oximetry, FSST = Foetal scalp stimulation test, MSAF = Meconium-staining amniotic fluid, NST = Non-stress test, rBPP = rapid Biophysical Profile, UADV = Umbilical artery Doppler velocity

*Pregnancy risk determination was based either: author’s specific mention of “low risk” and “high risk” pregnancies OR based on maternal and foetal risk factors described in the text. If no information available on maternal factors for “singleton, cephalic, >37” pregnancies the risk was status was defined as unclear.

**Table 3:** Foetal monitoring methods as predictors of birth outcomes.

Method			Predicts perinatal outcomes/foetal distress^1^	Improves perinatal outcomes^1^	Predicts mode of delivery	Increases operative deliveries	Improves Maternal morbidity/ mortality
Admission test		CTG(n = 7)					
		IA (Doppler, n = 1))					
		Admission AFI (n = 1)					
		FAST (n = 3)					
		rBPP(n = 1)					
		UADV(n = 2)					
Ongoing intrapartum foetal monitoring	FHR	IA Pinard(n = 6) REFERENCE^2^					
		IA Doppler(n = 3)					
		CTG(n = 11)					
		Partograph(n = 5)					
	Adjunctive tests	MSAF(n = 7)					
		FSST(n = 1)					
		FBS(n = 1)					
		FPO(n = 1)					
		UADV(n = 1)					

Green = Yes; Red = No; Orange = Unclear (i.e. outcome not reported or the evidence conflicts across studies). AFI = Amniotic fluid index, CTG = cardiotocograph, FAST = Foetal acoustic stimulation test, FBS = Foetal blood sampling, FPO = Foetal pulse oximetry, FSST = Foetal scalp stimulation test, MSAF = Meconium-staining amniotic fluid, rBPP = rapid Biophysical Profile, UADV = Umbilical artery Doppler velocity

^1^Perinatal outcomes any of the following: Apgar score at 1 or 5 minutes, umbilical cord blood pH/gases, need for neonatal resuscitation, stillbirth (intrapartum/fresh), neonatal deaths before discharge/within 24hours, admission to neonatal care unit, hypoxic-ischaemic encephalopathy

^2^Pinard was used as a reference test for which Doppler and CTG were compared to.

### Risk of bias of studies

A summary of quality assessment for intervention studies is provided in Table 1 and for observational studies in Table 2. Study performance of the five intervention studies was overall moderate, however, blinding of the participants or researchers was not done (5/5 high risk) and confounders were often not considered (1/5 high risk; 3/5 unclear risk and 1/5 low risk). Quality of observational studies was low to moderate; classified as low risk in: 81.3% for selection process, in 25% for comparability and in 25% outcome/exposure of studies.

### Narrative synthesis of quantitative results

A summary of the FHR monitoring strategies and their outcomes is provided in Table 3. Detailed results of each intervention and descriptive study are presented in S1–S3 Table.

### Admission tests

#### Neonatal outcomes

We identified only observational studies for admission tests. The study of IA on admission **(**n = 1) showed that absent FHR by hand-held Doppler was associated with a much higher perinatal mortality (938/1000 deliveries) compared to when FHR was present (13/1000 deliveries) [59].

Admission CTG, a 20-minute recording, was assessed in seven studies in Asia. Studies were on low risk (n = 3) [44,57,60], high risk (n = 3) [34,48,56], and mixed-risk pregnancies(n = 1) [32]. Abnormal CTG traces were associated with intrapartum FHR abnormalities (foetal distress) [32,34,44,48,57], meconium-stained liquor [32,57]; low Apgar scores at 5 minutes [32,44,48,57,60], perinatal deaths [32,44,48], and admission to neonatal intensive care unit (NICU) [32,44,48,57]. Test performance of admission CTG varied across studies: PPV of 19% to 88%, while the NPV was between 88.6% to 100% [32,48,56,57,60].

Maternal perception of sound-provoked foetal movement (i.e. foetal acoustic stimulation, FAST, n = 2) performed well in predicting foetal distress, perinatal death, Apgar score <7 at 5 minutes, admission to NICU [60,61]. It also improved the test performance of CTG in two studies (PPV: 45.2% to 65.5%, 19% to 73.6%, NPV: 94.2% to 96%, 100% to 100%) [56,60]. Admission amniotic fluid index (aAFI) performed worse than admission CTG (specificity: 64% and 92% respectively, n = 326) [35]. In one study (n = 330) rapid Biophysical profile (rBPP i.e. combination of sound-provoked ultrasound-detected foetal movement and AFI) had PPV (50%) and NPV (99.1%) for poor neonatal outcomes [51]. Umbilical artery Doppler velocity (UADV) in labour did not predict neonatal outcomes in two studies [33,49].

#### Maternal outcomes

Only studies on admission CTG reported mode of delivery. Abnormal traces increased CS rates compared to reactive CTG traces (between 42.7% to 100% and 20.7% to 30%, respectively, p<0.05) [32,34,44,57].

### Ongoing intrapartum foetal surveillance

#### Neonatal outcomes

There were three RCTs comparing IA and CTG: Uganda (n = 1971) [27], Zimbabwe (n = 1255) [26], India (n = 100) [28]. In Uganda, hand-held Doppler and Pinard stethoscope were compared [27]. The RCT in Zimbabwe had four arms: 1) intermittent CTG traces(n = 318) 2) hand-held Doppler (n = 312), 3) Pinard (n = 310)and 4) routine monitoring with Pinard (n = 315). In the first three groups, research midwives ensured they assessed FHR every 30 minutes for 10 minutes per study protocol and caregiving midwives were supposed to adhere to the same frequency by following hospital protocol [26]. Continuous CTG monitoring (n = 50) was compared to IA (n = 50) in women with a history of CS in India [28]. In these studies, detection of FHR abnormalities was significantly different in Pinard, Doppler and CTG groups (Table S1and S2). However, no significant changes in perinatal deaths, low Apgar scores at 1 and 5 minutes and admission to NICU were observed [26–28].

The study in Zimbabwe reported fewer cases of neonatal seizures and hypoxic-ischaemic encephalopathy (HIE) in the hand-held Doppler group compared to the Pinard groups (zero vs 15; and one vs 17 respectively) [26]. Although foetal distress was diagnosed in the three treatment groups, protocol violations, delays or unavailable operative deliveries led to the majority of perinatal deaths [26].

One observational study (Tanzania, n = 10271) showed that detection of an absent or abnormal FHR with foetal stethoscope was strongly associated with fresh stillbirths, neonatal deaths, low Apgar score and neonatal resuscitation [36]. In observational studies, pathological CTG traces were associated with low Apgar score at one minute [30,39], umbilical cord indices [30,50] and HIE [53] as compared to normal traces. However, contrasting findings were seen for umbilical cord indices (PPV 11.6% vs 100%) [43,50] and five minutes Apgar scores [30,37].

Several studies identified adjunctive tests for FHR monitoring. Foetal pulse oximetry had a comparable test performance compared to foetal blood sampling (n = 150) [42]. Meconium was mostly effective in predicting neonatal outcomes when combined with abnormal FHR [39,41,43,46]. Foetal scalp stimulation test (FSST) combined with IA were good predictors of perinatal outcomes: umbilical cord pH, Apgar score at one and five minutes, neonatal death and NICU admission [45].

The multi-centre partograph-intervention study in Southeast Asia which included 35 484 women showed a significant reduction in intrapartum stillbirths (0.50% to 0.31%, p = 0.024), but no significant reduction in Apgar scores, neonatal deaths, NICU, and resuscitation [24,25]. Training midwives to use the partograph reduced low Apgar scores at 1 minute but no improvement in other perinatal outcomes [29]. Observational studies showed that crossing the alert and action lines on the partograph was associated with a higher incidence of neonatal resuscitation and fresh stillbirths [54,55]. Substandard use of partograph was associated with low Apgar score [40].

#### Maternal outcomes

The RCT in Zimbabwe showed that CTG and hand-held Doppler significantly increased CS rates due to foetal distress compared to Pinard. (63%, 67% and 41% respectively) [26]. The RCT in India showed a trend towards increasing CS rate in the CTG group due to foetal distress compared to IA (47% vs 18%) [28]. The Uganda RCT showed no difference in overall CS rates between hand-held Doppler and Pinard [27]. No clear difference was observed for operative vaginal delivery [26,28]. Duration of labour [26]. postpartum haemorrhage, maternal fever, ruptured uterus and maternal death [28] were similar. Meconium was associated with increased CS rates in India (clear liquor 17% vs meconium 33%) [39,58]. Nonreactive FSST detected by IA was associated with a significant increase in operative vaginal deliveries and CS rates [45]. Two clustered RCT on the partograph showed that training and the use of partograph led to significant reduction in length of labour and obstructed labour and oxytocin use but no changes in CS rate or maternal mortality [24,25,29]. There was no increased CS rate due to foetal distress. There was a reduction in vaginal examinations but no change in postpartum haemorrhage and maternal sepsis.^27,33^ The partograph significantly increased the number of referrals of women in labour to higher level centres [29].

### Narrative synthesis of SWOT analysis

Detailed SWOT results of the given foetal monitoring methods are provided in Table 4. Admission CTG were recommended for triaging labours and resource allocation when resources are scarce [32,48,57]. The Pinard, hand-held Doppler and partograph were strategies reported as simple and low-cost [25–27,36,54,59]. IA allowed for greater mobility of the women than CTG and was easily accessible, but difficult to carry out in busy maternity wards [43]. The hand-held Doppler may be more mother- and user-friendly than the Pinard [27,59] but required consumables [36]. Some of these challenges were eliminated when using the wind-up Doppler. The use of CTG required a high level of skills, resources, and costs [26,31]. Combining FHR monitoring with simpler adjunctive tests such as meconium, FAST, FSST, and FPO may provide non-invasive and reliable ways to confirm foetal wellbeing, avoiding unnecessary interventions [42,45,56,60,61].

**Table 4:** SWOT analysis of methods of intrapartum foetal monitoring.

	Strengths	Weaknesses	Opportunities	Threats
**IA**	Detection of non-viable foetuses[36,59]	False results due to poor equipment [36]	Allows planning for safer delivery if intrauterine foetal death (on admission)[59]-Coupling of IA and partograph for monitoring[26,27,45] -Doppler may be preferred by care providers and pregnant women[26,27]	Limited human resources[36]
	Lower cost and sustainable[27]	Cannot detect subtle abnormalities or changes in FHR e.g. baseline variability[31,41,53]	Can be used as an intrapartum stillbirth indicator for monitoring quality improvement of care for interventions (on admission)[59] Can lead to prompt emergency obstetric and neonatal care obstetrical[36]	Not always used on admission/intrapartum[36,59]
	Can detect ir/regular rhythms, accelerations and decelerations[26,27,31,41]	Difficult to use, time-consuming and labour intensive[27,36]	Training may improve performance[59]	False results due poorly trained staff[36,46,59]
	Allows mobility of the women[31]	Uncomfortable for the mother and staff (Pinard)[26,36]	Promotes ‘‘hands-on” support to the labouring woman[31]	Lack of foetal monitoring protocol[46]
	Requires no additional resources/electricity (Pinard/wind-up Doppler)[27,36] *Hand-held Doppler*: Gives a steady number of beats per minute[26,27]	Maternal heart rate may occasionally be counted[26]		Non-adherence to frequency, duration of monitoring and documentation[31] Underutilisation of partograph[27,40]
	Device easy to use with minimal training[26,36,59]			Delays in action taking (long diagnosis to delivery time) [26,27,31]
	Audible to both mother and caregiver (even in noisy labour wards) [27]			Unavailability of operative delivery[26]
				Unavailability of FBS and cord blood analysis to confirm foetal compromise[26,27]
				May require repair and additional resources (Doppler)[26,27]
				Responsible of large proportion of CS are due to suspected foetal distress[31,41]
**CTG**	Non-invasive(external) [26,28,30,34,37–39,43,44,47,48,50,53,56–58,60] -Continuous traces of FHR [26,28,30,34,37–39,43,44,47,48,50,53,56–58,60]	Associated with high false positivity for foetal distress[26,28,30,34,37–39,43,44,47,48,50,53,56–58,60] -Admission CTG might not predict foetal distress several hours after admission. [32,48]	Can be used intermittently during labour [26]	Potential increase in unnecessary interventions (e.g. caesarean section)[30,34,38,39,47,50]
	Able to detect subtle changes in FHR e.g. baseline variability [26,28,30,34,37–39,43,44,47,48,50,53,56–58,60]	Low inter-observer agreement[47]	*Admission test*: Screening test for foetal distress on admission[32,34,44,48]	Costly and requires maintenance [26]
	Several pathological features are predictive of foetal acidosis [26,28,30,34,37–39,43,44,47,48,50,53,56–58,60]	Susceptible to technical and mechanical failure resulting in poor quality of traces and interpretation[26]	*Admission test*: Prevent unnecessary delay in intervention[32]	Non-adherence of staff to protocol[26]
			*Admission test*: Triaging: allows selection of patients for closer monitoring during limited resources[32,34,44,48]	Limited or unavailability of CTG machine[31,34,41]
				Delays in action taking (long diagnosis to delivery time) [26]
				No facility to perform FBS[41,50]
				Unstable electricity supply[31]
				Medicolegal climate[47]
				Contraction may impair maternal perception of foetal movement[61]
**Foetal stimulation tests**	Non-/less- invasive[56]	Poor maternal perception of subtle foetal movement[60,61]	Safer to use in over-distended and scarred uterus [60]	
	Fast, simple and cheaper[45,60,61]		Can be used to increase diagnostic accuracy of FHR monitoring: IA [45] or EFM[32,34,48,56,60]and MSAF[45] as an alternative to FBS[45,61]	
	No additional device necessary (scalp stimulation)[45]		Screening tool in early labour[60,61]	
	No rupture of membranes required[45]			
**rBPP**	Simple and fast[51]	Not adequate as a screening test[51]	May be used as an additional back up test[51]	
	Relatively inexpensive[51]			
**UADV**	Feasible and no discomfort in labour[33]	Not useful in detecting foetal acidosis during labour[49]		
	Non-invasive and simple[33,49]			
**MSAF**	A warning sign that closer attention is warranted[58]	Highly unreliable when used alone[43,45]	More reliable when combined with FHR monitoring (IA [31,41,46] and CTG[39,43,58])	Association with an increase in caesarean[39,58]
		Require ruptured membranes[39]		
**FBS** **and FPO**	FPO is less invasive than FBS[42]	Recordings take 30 minutes (time-consuming)[42]	May decrease unnecessary interventions (e.g. CS) [42]	
			FPO may be an alternative to foetal blood sampling[42]	
**Partograph**	Provides recording of the foetal and maternal parameters[25,29]	Too detailed[40]	Encourages supportive care to women [24]	Incorrect and/ incompletion of partographs: e.g. due to lack of time, motivation, human resources[24,29,40,54]
	Single page[55]	Requires intensive and repeated training[40]	Helps interpret findings[40]	Loss of partographs[54]
	Visual presentation with clear overview of progress of labour[55]	Applicable mostly in first stage of labour[25,54,55]	Training and supervision improves use[29,54]	The need for photocopying[40]
	Accompanied by management protocol[25]		Helps communication and hand-over of patients between staff [25,40]	Lack of updated versions[40]
			Permits evaluation of quality of care[40]	Removal of latent phase causes incomplete follow-up and difficulty in diagnosing prolonged latent phase[29]
			Timely referral[29]	Unavailability of guidelines in labour wards[40]
			Early diagnosis of complications and early decision making[40]	Non-adherence to protocol[29]
			Labour wards can opt for adapted local management protocols[25]	Lack of training and supervision[40]
			Universal application[25,40]	Lack of appropriate intervention[26,27]
				High rates of referral[25,29]

CTG = cardiotography, CS = caesarean, IA = Intermittent Auscultation, FBS = Foetal blood sampling, FHR = Foetal heart rate, FPO = Foetal pulse oximetry, MSAF = Meconium-staining amniotic fluid, rBPP = Rapid biophysical profile, SWOT = Strengths, Weaknesses, Opportunities, Threats, UADV = Umbilical artery Doppler velocity.

Strengths of the partograph were its low-cost, pictorial overview of labour allowing timely recognition for complications and action [25,29,40,54,55]. A major threat was an underuse of partograph due to a shortage of staff, lack of knowledge, training, and guidelines, unavailability of copies and hesitant attitudes of staff [29,40,54]. Opportunities to increase partograph use lie in providing partograph copies, training, and appropriate management guidelines [25,29,40,54]. A major threat to all intrapartum foetal surveillance studies was limited or unavailability of intervention including timely operative deliveries [26,27,31,36].

## Discussion

### Main findings

This systematic review and SWOT analysis provide an overview of the evidence of intrapartum foetal monitoring strategies in low-resource settings on perinatal and maternal outcomes. The use of CTG increased the rates of CS but had no effect on adverse perinatal outcomes compared to IA [26,28]. IA and the partograph is the preferred method in low-resource settings for FHR monitoring.

The observational studies in this review suggest that admission tests (including CTG, IA or FAST) can predict adverse outcomes in LMICs, and mode of delivery in both low and high-risk pregnancies [32,34,48,56,57,60,61]. We suggest that admission tests might have a much better use in low resource settings because of: 1) the incidence of intrapartum stillbirths could modify the predictive test results [11], 2) inadequate risk assessment and stratification during antenatal care, making admission tests a good screening tool to identify high-risk foetuses and 3) a triaging tool for better allocation of resources in settings with heavy workload and scarce (human) resources [32,34,48,57].

The overall evidence shows that CTG does not improve outcomes but increases the number of CS compared to IA. It is unclear whether hand-held Doppler improves neonatal outcomes, and it may increase CS rate. Similar findings on CTG and hand-held Doppler are reported in the Cochrane meta-analyses [11,62]. A study in South Africa showed pregnant women preferred hand-held Doppler over Pinard or CTG [63]. However, the number of CS presents real concerns for maternal safety in low resource settings [64–68]. Foetal heart monitoring may have false positivity for foetal distress leading to unnecessary intervention. The current review identified simple and cheap strategies to strengthen the test performance of intrapartum FHR monitoring including foetal stimulation tests (FAST and FSST) and meconium. However, their effectiveness is not known and should be tested in future studies. Contrary to a Cochrane review, which did not include the large study in South East Asia [69], the partograph was useful for monitoring and decision-making for the intrapartum care of the mother, foetus and labour progress, and was associated with reduced intrapartum stillbirths in low-resource settings [25,29,40,54,55]. The BOLD initiative and WHO guidelines stress the importance of supportive, person-centred care during labour and childbirth rather than focus on cervical dilatation only [70–72].

Challenges exist in up-scaling effective interventions in low-resource settings [18,73]. Given the resource constraints, the SWOT analysis shows that the ideal method of intrapartum foetal monitoring should be: simple, affordable, robust, safe, reliable and sustainable [18,74]. Yet, most monitoring systems require maintenance and adequate staffing who need to be trained and supervised. For example, although IA and partographs are low-tech and -cost technology, they highly depend on human resources. A strong commitment to investing in high quality research of existing and new strategies of real-life implementation for intrapartum foetal monitoring is required. These may include new ways to monitor foetal well-being, context-appropriate guidelines, and healthcare workforce strengthening [15,75]. A substantial time-lag between recognition of foetal compromise and delivery as a major cause of severe asphyxia and death was identified in this review [26,27,31,36]. Importantly, emergency obstetric and newborn care including operative vaginal deliveries and neonatal resuscitation should be readily available to ensure both prompt diagnosis and successive intervention.

### Strengths and limitations

A strength of this review is the systematic assessment of neonatal and maternal outcomes and SWOT analysis. Although an extensive and inclusive search in five international databases was conducted, studies performed in low-resource settings and published in national journals might not have been indexed in the searched databases. Limitations are also inherent in the reviewed articles and include the quality of the evidence, the lack of detailed reporting of implementation factors and relevant outcomes such as contraction monitoring, maternal morbidity and mortality, CS rates, professional and maternal opinion. RCTs did not guarantee appropriate and timely interventions which confounded the results. We intended to evaluate evidence for all intrapartum foetal monitoring strategies in low-resource settings using a meta-analysis, however, due to heterogeneity in designs and outcomes, only a narrative review could be performed.

### Conclusion

Of the foetal monitoring strategies that have been studied in LMICs, the partograph and intermittent auscultation is the preferred strategy for intrapartum foetal surveillance in low-resource settings because of reduced intrapartum stillbirths (partograph), lower caesarean section rates (Pinard) and easier implementation. CTG is associated with higher caesarean section rates without proven benefits for perinatal outcomes, and should not be recommended in low-resource settings until new research delivers evidence for better perinatal outcomes. The benefit and harms of admission tests, adjunctive tests and hand-held Doppler on perinatal and maternal outcomes should be determined in future studies in low resource settings. High-quality RCT studies of foetal monitoring should include clear management protocols with timely interventions. Moreover, there is a need to harmonise core outcomes in foetal monitoring studies. Consideration of implementation factors will also be essential to determine the real-world optimal foetal monitoring approach.

## Supporting information

S1 File PRISMA 2009 checklist. (DOCX)

S2 File Full search strategy for five databases and data extraction sheet. (DOCX)

S3 File The Cochrane risk of bias tool. (PDF)

S4 File New Castle-Ottawa scale for cohort and case-control studies. (PDF)

S5 File New Castle-Ottawa scale cross-sectional studies. (DOC)

S1 Table Summary of quantitative results. (DOCX)

S2 Table Characteristics and quantitative results of included randomised controlled trials. (DOCX)

S3 Table Characteristics and quantitative results of included observational studies. (DOCX)
