# Supplementary material for: Strategies for intrapartum foetal surveillance in low- and middle-income countries: A systematic review
Source: PLoS One. 2018 Oct 26;13(10):e0206295. doi: 10.1371/journal.pone.0206295 (PMC6203373; doi:10.1371/journal.pone.0206295)
Supplement: S2 File — (DOCX) [file pone.0206295.s002.docx]

**Appendix A: Search Strategy**

**Search string Pubmed**

| **P** | Intrapartum | “labor, obstretric”[Mesh] OR intrapartu*[Title/Abstract] OR childbirth*[Title/abstract] OR child birth[Title/abstract] OR delivery[Title/Abstract] OR labour*[Title/Abstract) OR labor*[Title/abstract] |
| --- | --- | --- |
| **I** | Foetal surveillance | "Fetal Monitoring"[Mesh] OR “Heart rate, fetal”[Mesh] OR fetal monitoring*[Title/Abstract] OR foetal monitoring*[Title/Abstract] OR ((fetal[Title/Abstract] OR foetal[Title/Abstract] OR unborn child[Title/abstract]) AND surveillance[Title/Abstract]) OR ((fetal[Title/Abstract] OR foetal[Title/Abstract] OR unborn child[Title/abstract]) AND assessment*[Title/Abstract]) OR ((fetal[Title/Abstract] OR foetal[Title/Abstract] or unborn child[Title/abstract]) AND stimulation[Title/abstract] AND (test[Title/abstract] OR tests[Title/abstract] or testing[Title/abstract])) OR ((fetal[Title/Abstract] OR foetal[Title/Abstract]) AND (well-being* [Title/abstract] OR wellbeing* [Title/Abstract])) OR ((fetal[Title/abstract] or foetal[Title/abstract]) AND heart rate[Title/abstract] AND monitoring*[Title/abstract]) OR fetus supervision*[Title/abstract] OR foetus supervision*[Title/abstract] OR foetal supervision*[Title/abstract] OR fetal supervision*[Title/abstract] OR ((fetal[Title/abstract] OR foetal[Title/abstract]) AND heart[Title/abstract] AND (test[Title/abstract] OR tests[Title/abstract] OR testing[Title/abstract] OR tested[Title/abstract])) OR fetal heart tracing*[Title/abstract] OR foetal heart tracing*[Title/abstract] OR fetal testing[Title/abstract] OR foetal testing[Title/abstract] OR ((foetal[Title/abstract] OR fetal[Title/abstract]) AND condition predictor[Title/abstract]) OR (intrapartum[Title/abstract] AND (test[Title/abstract] OR tests[Title/abstract] OR testing[Title/abstract])) |
| **C** | - | - |
| **O** | Outcomes | "Perinatal Mortality"[Mesh] OR "Fetal Distress"[Mesh] OR ((Neonatal [Title/abstract] OR perinatal [Title/abstract] OR newborn[Title/abstract] OR fetal[Title/abstract] OR intrapartum-related[Title/abstract] OR intrauterine[Title/abstract] OR intra uterine[Title/abstract]) AND (outcome*[Title/Abstract] OR loss*[Title/abstract] OR morbidity[Title/abstract] OR mortality[Title/abstract] OR death*[Title/abstract] OR trauma[Title/abstract] OR resuscitation*[Title/Abstract] OR seizure*[Title/Abstract] OR distress*[Title/abstract] OR complication*[Title/abstract] OR acidemia[Title/abstract])) OR fetal wellbeing[Title/abstract] OR ((neonatal[Title/abstract] OR NICU[Title/abstract]) AND admission*[Title/Abstract]) OR apgar[Title/Abstract] OR asphyxia[Title/Abstract] OR cerebral palsy[Title/Abstract] OR encephalopathy[Title/Abstract] OR acidosis[Title/Abstract] OR stillbirth[Title/Abstract] OR stillborn[Title/Abstract] OR disability[Title/Abstract] OR ((developmental[Title/abstract] OR neurodevelopmental[Title/abstract]) AND (disability [Title/Abstract] OR delay[Title/abstract])) OR meconium aspiration [Title/Abstract] OR cognitive function[Title/Abstract] OR mode of birth[Title/abstract] OR ((cesarean[Title/Abstract] OR caesarean[Title/abstract]) AND section*[Title/abstract]) OR ((instrumental[Title/abstract] OR spontaneous[Title/abstract]) AND delivery[Title/abstract]) OR blood gas [Title/abstract] OR assisted ventilation[Title/abstract] OR ventilator support[Title/abstract] |
|  | NOT | cat OR cats OR dog OR dogs OR cattle OR animal OR animals OR rat OR rats OR mice OR mouse OR sheep OR horse OR horses OR bovine OR lamb OR lambs OR foals OR foal OR ovine OR gorilla* OR rabbit* OR baboon* OR goat OR goats OR elephant* OR monkey* OR cytomegalovirus OR cholestasis OR anesthesia OR (down’s syndrome) OR cystic fibrosis OR spina bifida OR cancer OR congenital heart disease OR SLE OR cardiac surgery |

| Combinations | Number of Articles | Details |
| --- | --- | --- |
| Foetale surveillance AND intrapartum | 9125 | Search in title/abstract |
| Foetale surveilance AND intrapartum AND outcomes | …. | Search in title/abstract |

- cat OR cats OR dog OR dogs OR cattle OR animal OR animals OR rat OR rats OR mice OR mouse OR sheep OR horse OR horses OR bovine OR lamb OR lambs OR foals OR foal OR ovine OR gorilla OR rabbit* OR baboon* OR goat OR goats OR elephant* OR monkey* (217), cytomegalovirus (7), cholestasis (38), anesthesia (155),
- down’s syndrome (6), cystic fibrosis (7), spina bifida(7), cancer (24)
- congenital heart disease (41), SLE (19)

**Search string EMBASE**

| Intrapartum | 'childbirth'/exp OR 'intrapartum care'/exp OR intrapartum*:ab,ti OR childbirth*:ab,ti OR delivery*:ab,ti OR labour*:ab,ti OR laboring*:ab,ti |
| --- | --- |
| Foetal surveillance | 'fetus control'/exp OR 'fetal well being'/exp OR 'fetus heart rate'/exp OR ((fetal:ab,ti OR foetal:ab,ti) AND monitoring*:ab,ti) OR (fetal:ab,ti AND surveillance:ab,ti) OR (foetal:ab,ti AND surveillance:ab,ti) OR (fetal:ab,ti AND assessment*:ab,ti) OR (foetal:ab,ti AND assessment*:ab,ti) OR (fetal:ab,ti AND stimulation:ab,ti AND (test:ab,ti OR testing:ab,ti OR tests:ab,ti)) OR (foetal:ab,ti AND stimulation:ab,ti AND (test:ab,ti OR testing:ab,ti OR tests:ab,ti)) OR (fetal:ab,ti AND ('well being':ab,ti OR ‘wellbeing’:ab,ti)) OR (foetal:ab,ti AND ('well being':ab,ti OR ‘wellbeing’:ab,ti)) OR (fetal:ab,ti AND heart:ab,ti AND rate:ab,ti AND monitoring*:ab,ti) OR (foetal:ab,ti AND heart:ab,ti AND rate:ab,ti AND monitoring*:ab,ti) OR (fetus:ab,ti AND supervision*:ab,ti) OR (foetus:ab,ti AND supervision*:ab,ti) OR (foetal:ab,ti AND supervision*:ab,ti) OR (fetal:ab,ti AND supervision*:ab,ti) OR (fetal:ab,ti AND heart:ab,ti AND (test:ab,ti OR testing:ab,ti OR tests:ab,ti)) OR (foetal:ab,ti AND heart:ab,ti AND (test:ab,ti OR testing:ab,ti OR tests:ab,ti)) OR (fetal:ab,ti AND heart:ab,ti AND tracing*:ab,ti) OR (foetal:ab,ti AND heart:ab,ti AND tracing*:ab,ti) OR ((foetal:ab,ti OR fetal:ab,ti) AND testing:ab,ti) OR ((foetal:ab,ti OR fetal:ab,ti) AND condition:ab,ti AND predictor:ab,ti) OR (intrapartum:ab,ti AND (test:ab,ti OR tests:ab,ti OR testing:ab,ti)) |
| Outcome | ‘perinatal morbidity’/exp OR ‘perinatal mortality’/exp OR ‘fetus distress’/exp OR ‘fetal well being’/exp OR ((Neonatal:ab,ti OR perinatal:ab,ti OR newborn:ab,ti OR fetal:ab,ti OR intrapartum-related:ab,ti OR intrauterine:ab,ti OR (intra:ab,ti AND uterine:ab,ti) OR ‘unborn child’:ab,ti) AND (outcome*:ab,ti OR loss*:ab,ti OR morbidity:ab,ti OR mortality:ab,ti OR death*:ab,ti OR trauma:ab,ti OR resuscitation*:ab,ti OR seizure*:ab,ti OR complication*:ab,ti)) OR ((neonatal:ab,ti OR NICU:ab,ti) AND admission*:ab,ti) OR apgar:ab,ti OR asphyxia:ab,ti OR cerebral palsy:ab,ti OR encephalopathy:ab,ti OR acidosis:ab,ti OR stillbirth:ab,ti OR stillborn:ab,ti OR disability:ab,ti OR ((developmental:ab,ti OR neurodevelopmental:ab,ti) AND (disability:ab,ti OR delay:ab,ti)) OR (meconium:ab,ti AND aspiration:ab,ti) OR (cognitive:ab,ti AND function:ab,ti) OR (mode:ab,ti AND birth:ab,ti) OR ((cesarean:ab,ti OR caesarean:ab,ti) AND section*:ab,ti) OR ((instrumental:ab,ti OR spontaneous:ab,ti) AND delivery:ab,ti) OR (blood:ab,ti AND gas:ab,ti) OR (assisted:ab,ti AND ventilation:ab,ti) OR (ventilator:ab,ti AND support:ab,ti) OR (fetal:ab,ti AND wellbeing:ab,ti) |

| Input | Number of articles | Details |
| --- | --- | --- |
| Intrapartum | 479359 |  |
| Foetal surveillance | 63092 |  |
| Intrapartum AND Foetal surveillance | 16559 | No limits used |
| Intrapartum AND Foetal surveillance AND outcome | 6068 | No limits used |

**Search string Cochrane library**

| Intrapartum | labor*:**ti,ab** OR intrapartu****:ti,ab*** OR childbirth*****:ti,ab*** OR delivery****:ti,ab*** OR labour****:ti,ab*** |
| --- | --- |
| Foetal monitoring | fetal monitoring:ti,ab or foetal monitoring:ti,ab or fetal surveillance*:ti,ab or foetal surveillance:ti,ab or fetal assessment*:ti,ab or foetal assessment*:ti,ab or fetal stimulation test*:ti,ab or foetal stimulation test*:ti,ab or fetal well-being:ti,ab or foetal well-being:ti,ab or fetal wellbeing:ti,ab or foetal wellbeing:ti,ab or fetal heart rate monitoring*:ti,ab or foetal heart rate monitoring*:ti,ab or fetus supervision*:ti,ab or foetus supervision*:ti,ab or foetal supervision*:ti,ab or fetal supervision*:ti,ab or fetal heart test:ti,ab or foetal heart test:ti,ab or fetal heart tests:ti,ab or foetal heart tests:ti,ab or fetal heart testing:ti,ab or foetal heart testing:ti,ab or foetal heart tracing*:ti,ab or fetal heart tracing*:ti,ab or fetal testing:ti,ab or foetal testing:ti,ab or foetal condition predictor:ti,ab or fetal condition predictor:ti,ab or intrapartum test:ti,ab or intrapartum tests:ti,ab or intrapartum testing:ti,ab |
|  |  |

| Input | Number of articles | Details |
| --- | --- | --- |
| Intrapartum | 45954 |  |
| Foetal surveillance | 1795 |  |
| Intrapartum AND Foetal surveillance | 1064 | No Search limit |

**POPline search**

| Intrapartum | (labor*) OR (intrapartu*) OR (childbirth*) OR (delivery) OR (labour*) |
| --- | --- |
| Foetal monitoring | (fetal monitoring*) OR (foetal monitoring*) OR (fetal surveillance*) OR (foetal surveillance) OR (fetal assessment*) OR (foetal assessment*) OR (fetal stimulation test*) OR (foetal stimulation test*) OR (fetal well-being*) OR (foetal well-being*) OR (fetal wellbeing) OR (foetal wellbeing) OR (fetal heart rate monitoring*) OR (foetal heart rate monitoring*) OR (fetus supervision*) OR (foetus supervision*) OR (foetal supervision*) OR (fetal supervision*) OR (fetal heart test*) OR (foetal heart test*) OR (fetal heart tracing*) OR (foetal heart tracing*) OR (fetal testing) OR (foetal testing) OR (foetal condition predictor) OR (fetal condition predictor) OR (intrapartum test) OR (intrapartum tests) OR (intrapartum testing) |

| Combination | Number of articles | Search strategy |
| --- | --- | --- |
| Intrapartum AND Foetal monitoring | 800 | All fields |
| Intrapartum AND Foetal monitoring | 3 | Title |

NB: ((fetal monitoring*) OR (foetal monitoring*) OR (fetal surveillance) OR (foetal surveillance*) OR (fetal assessment*) OR (foetal assessment*) OR (fetal stimulation test*) OR (foetal stimulation test*) OR (fetal well-being*) OR (foetal well-being*) OR (fetal heart rate monitoring*) OR (foetal heart rate monitoring*) OR (fetus supervision*) OR (foetus supervision*) OR (foetal supervision*) OR (fetal supervision*) OR (fetal heart test*) OR (foetal heart test*) OR (fetal heart tracing*) OR (foetal heart tracing*) OR (fetal testing) OR (foetal testing)) AND ((labor*) OR (intrapartu*) OR (childbirth*) OR (delivery) OR (labour*))

**Global Health Library Search**

| Intrapartum | (labor*) OR (intrapartu*) OR (childbirth*) OR (delivery) OR (labour*) |
| --- | --- |
| Foetal monitoring | (fetal monitoring*) OR (foetal monitoring*) OR (fetal surveillance) OR (foetal surveillance) OR (fetal assessment*) OR (foetal assessment*) OR (fetal stimulation test*) OR (foetal stimulation test*) OR (fetal well-being) OR (fetal wellbeing) OR (foetal wellbeing) OR (foetal well-being) OR (fetal heart rate monitoring*) OR (foetal heart rate monitoring*) OR (fetus supervision*) OR (foetus supervision*) OR (foetal supervision*) OR (fetal supervision*) OR (fetal heart test*) OR (foetal heart test*) OR (fetal heart tracing*) OR (foetal heart tracing*) OR (fetal testing) OR (foetal testing) OR (foetal condition predictor) OR (fetal condition predictor) OR (intrapartum test) OR (intrapartum tests) OR (intrapartum testing) |

| Combination | Number of articles | Search strategy |
| --- | --- | --- |
| Intrapartum AND foetal monitoring | 59 | Title, abstract and subject  Filter to leave out Medline  Limit to humans  English only |

NB: (tw:((fetal monitoring*) OR (foetal monitoring*) OR (fetal surveillance) OR (foetal surveillance) OR (fetal assessment*) OR (foetal assessment*) OR (fetal stimulation test*) OR (foetal stimulation test*) OR (fetal well-being*) OR (foetal well-being*) OR (fetal heart rate monitoring*) OR (foetal heart rate monitoring*) OR (fetus supervision*) OR (foetus supervision*) OR (foetal supervision*) OR (fetal supervision*) OR (fetal heart test*) OR (foetal heart test*) OR (fetal heart tracing*) OR (foetal heart tracing*) OR (fetal testing) OR (foetal testing))) AND (tw:((labor*) OR (intrapartu*) OR (childbirth*) OR (delivery) OR (labour*))) AND (instance:"ghl") AND ( db:("LILACS") AND limit:("humans") AND la:("en"))

LMIC search string

**#10 Search** #1 or #2 or #3 or #4 or #5 or #6 or #7 or #8 or #9

**#9 Search** "developing country"[tiab] OR "developing countries"[tiab] OR "developing nation"[tiab] OR "developing nations"[tiab] OR "developing population"[tiab] OR "developing populations"[tiab] OR "developing world"[tiab] OR "less developed country"[tiab] OR "less developed countries"[tiab] OR "less developed nation"[tiab] OR "less developed nations"[tiab] OR "less developed population"[tiab] OR "less developed populations"[tiab] OR "less developed world"[tiab] OR "lesser developed country"[tiab] OR "lesser developed countries"[tiab] OR "lesser developed nation"[tiab] OR "lesser developed nations"[tiab] OR "lesser developed population"[tiab] OR "lesser developed populations"[tiab] OR "lesser developed world"[tiab] OR "under developed country"[tiab] OR "under developed countries"[tiab] OR "under developed nation"[tiab] OR "under developed nations"[tiab] OR "under developed population"[tiab] OR "under developed populations"[tiab] OR "under developed world"[tiab] OR "underdeveloped country"[tiab] OR "underdeveloped countries"[tiab] OR "underdeveloped nation"[tiab] OR "underdeveloped nations"[tiab] OR "underdeveloped population"[tiab] OR "underdeveloped populations"[tiab] OR "underdeveloped world"[tiab] OR "middle income country"[tiab] OR "middle income countries"[tiab] OR "middle income nation"[tiab] OR "middle income nations"[tiab] OR "middle income population"[tiab] OR "middle income populations"[tiab] OR "low income country"[tiab] OR "low income countries"[tiab] OR "low income nation"[tiab] OR "low income nations"[tiab] OR "low income population"[tiab] OR "low income populations"[tiab] OR "lower income country"[tiab] OR "lower income countries"[tiab] OR "lower income nation"[tiab] OR "lower income nations"[tiab] OR "lower income population"[tiab] OR "lower income populations"[tiab] OR "underserved country"[tiab] OR "underserved countries"[tiab] OR "underserved nation"[tiab] OR "underserved nations"[tiab] OR "underserved population"[tiab] OR "underserved populations"[tiab] OR "underserved world"[tiab] OR "under served country"[tiab] OR "under served countries"[tiab] OR "under served nation"[tiab] OR "under served nations"[tiab] OR "under served population"[tiab] OR "under served populations"[tiab] OR "under served world"[tiab] OR "deprived country"[tiab] OR "deprived countries"[tiab] OR "deprived nation"[tiab] OR "deprived nations"[tiab] OR "deprived population"[tiab] OR "deprived populations"[tiab] OR "deprived world"[tiab] OR "poor country"[tiab] OR "poor countries"[tiab] OR "poor nation"[tiab] OR "poor nations"[tiab] OR "poor population"[tiab] OR "poor populations"[tiab] OR "poor world"[tiab] OR "poorer country"[tiab] OR "poorer countries"[tiab] OR "poorer nation"[tiab] OR "poorer nations"[tiab] OR "poorer population"[tiab] OR "poorer populations"[tiab] OR "poorer world"[tiab] OR "developing economy"[tiab] OR "developing economies"[tiab] OR "less developed economy"[tiab] OR "less developed economies"[tiab] OR "lesser developed economy"[tiab] OR "lesser developed economies"[tiab] OR "under developed economy"[tiab] OR "under developed economies"[tiab] OR "underdeveloped economy"[tiab] OR "underdeveloped economies"[tiab] OR "middle income economy"[tiab] OR "middle income economies"[tiab] OR "low income economy"[tiab] OR "low income economies"[tiab] OR "lower income economy"[tiab] OR "lower income economies"[tiab] OR "low gdp"[tiab] OR "low gnp"[tiab] OR "low gross domestic"[tiab] OR "low gross national"[tiab] OR "lower gdp"[tiab] OR "lower gnp"[tiab] OR "lower gross domestic"[tiab] OR "lower gross national"[tiab] OR lmic[tiab] OR lmics[tiab] OR "third world"[tiab] OR "lami country"[tiab] OR "lami countries"[tiab] OR "transitional country"[tiab] OR "transitional countries"[tiab]

**#8 Search** "developing country"[ot] OR "developing countries"[ot] OR "developing nation"[ot] OR "developing nations"[ot] OR "developing population"[ot] OR "developing populations"[ot] OR "developing world"[ot] OR "less developed country"[ot] OR "less developed countries"[ot] OR "less developed nation"[ot] OR "less developed nations"[ot] OR "less developed population"[ot] OR "less developed populations"[ot] OR "less developed world"[ot] OR "lesser developed country"[ot] OR "lesser developed countries"[ot] OR "lesser developed nation"[ot] OR "lesser developed nations"[ot] OR "lesser developed population"[ot] OR "lesser developed populations"[ot] OR "lesser developed world"[ot] OR "under developed country"[ot] OR "under developed countries"[ot] OR "under developed nation"[ot] OR "under developed nations"[ot] OR "under developed population"[ot] OR "under developed populations"[ot] OR "under developed world"[ot] OR "underdeveloped country"[ot] OR "underdeveloped countries"[ot] OR "underdeveloped nation"[ot] OR "underdeveloped nations"[ot] OR "underdeveloped population"[ot] OR "underdeveloped populations"[ot] OR "underdeveloped world"[ot] OR "middle income country"[ot] OR "middle income countries"[ot] OR "middle income nation"[ot] OR "middle income nations"[ot] OR "middle income population"[ot] OR "middle income populations"[ot] OR "low income country"[ot] OR "low income countries"[ot] OR "low income nation"[ot] OR "low income nations"[ot] OR "low income population"[ot] OR "low income populations"[ot] OR "lower income country"[ot] OR "lower income countries"[ot] OR "lower income nation"[ot] OR "lower income nations"[ot] OR "lower income population"[ot] OR "lower income populations"[ot] OR "underserved country"[ot] OR "underserved countries"[ot] OR "underserved nation"[ot] OR "underserved nations"[ot] OR "underserved population"[ot] OR "underserved populations"[ot] OR "underserved world"[ot] OR "under served country"[ot] OR "under served countries"[ot] OR "under served nation"[ot] OR "under served nations"[ot] OR "under served population"[ot] OR "under served populations"[ot] OR "under served world"[ot] OR "deprived country"[ot] OR "deprived countries"[ot] OR "deprived nation"[ot] OR "deprived nations"[ot] OR "deprived population"[ot] OR "deprived populations"[ot] OR "deprived world"[ot] OR "poor country"[ot] OR "poor countries"[ot] OR "poor nation"[ot] OR "poor nations"[ot] OR "poor population"[ot] OR "poor populations"[ot] OR "poor world"[ot] OR "poorer country"[ot] OR "poorer countries"[ot] OR "poorer nation"[ot] OR "poorer nations"[ot] OR "poorer population"[ot] OR "poorer populations"[ot] OR "poorer world"[ot] OR "developing economy"[ot] OR "developing economies"[ot] OR "less developed economy"[ot] OR "less developed economies"[ot] OR "lesser developed economy"[ot] OR "lesser developed economies"[ot] OR "under developed economy"[ot] OR "under developed economies"[ot] OR "underdeveloped economy"[ot] OR "underdeveloped economies"[ot] OR "middle income economy"[ot] OR "middle income economies"[ot] OR "low income economy"[ot] OR "low income economies"[ot] OR "lower income economy"[ot] OR "lower income economies"[ot] OR "low gdp"[ot] OR "low gnp"[ot] OR "low gross domestic"[ot] OR "low gross national"[ot] OR "lower gdp"[ot] OR "lower gnp"[ot] OR "lower gross domestic"[ot] OR "lower gross national"[ot] OR lmic[ot] OR lmics[ot] OR "third world"[ot] OR "lami country"[ot] OR "lami countries"[ot] OR "transitional country"[ot] OR "transitional countries"[ot]

**#7 Search** Africa[pl] OR Asia[pl] OR Caribbean[pl] OR West Indies[pl] OR South America[pl] OR Latin America[pl] OR Central America[pl] OR Afghanistan[pl] OR Albania[pl] OR Algeria[pl] OR Angola[pl] OR Antigua[pl] OR Barbuda[pl] OR Argentina[pl] OR Armenia[pl] OR Armenian[pl] OR Aruba[pl] OR Azerbaijan[pl] OR Bahrain[pl] OR Bangladesh[pl] OR Barbados[pl] OR Benin[pl] OR Byelarus[pl] OR Byelorussian[pl] OR Belarus[pl] OR Belorussian[pl] OR Belorussia[pl] OR Belize[pl] OR Bhutan[pl] OR Bolivia[pl] OR Bosnia[pl] OR Herzegovina[pl] OR Hercegovina[pl] OR Botswana[pl] OR Brasil[pl] OR Brazil[pl] OR Bulgaria[pl] OR Burkina Faso[pl] OR Burkina Fasso[pl] OR Upper Volta[pl] OR Burundi[pl] OR Urundi[pl] OR Cambodia[pl] OR Khmer Republic[pl] OR Kampuchea[pl] OR Cameroon[pl] OR Cameroons[pl] OR Cameron[pl] OR Camerons[pl] OR Cape Verde[pl] OR Central African Republic[pl] OR Chad[pl] OR Chile[pl] OR China[pl] OR Colombia[pl] OR Comoros[pl] OR Comoro Islands[pl] OR Comores[pl] OR Mayotte[pl] OR Congo[pl] OR Zaire[pl] OR Costa Rica[pl] OR Cote d'Ivoire[pl] OR Ivory Coast[pl] OR Croatia[pl] OR Cuba[pl] OR Cyprus[pl] OR Czechoslovakia[pl] OR Czech Republic[pl] OR Slovakia[pl] OR Slovak Republic[pl] OR Djibouti[pl] OR French Somaliland[pl] OR Dominica[pl] OR Dominican Republic[pl] OR East Timor[pl] OR East Timur[pl] OR Timor Leste[pl] OR Ecuador[pl] OR Egypt[pl] OR United Arab Republic[pl] OR El Salvador[pl] OR Eritrea[pl] OR Estonia[pl] OR Ethiopia[pl] OR Fiji[pl] OR Gabon[pl] OR Gabonese Republic[pl] OR Gambia[pl] OR Gaza[pl] OR Georgia Republic[pl] OR Georgian Republic[pl] OR Ghana[pl] OR Gold Coast[pl] OR Greece[pl] OR Grenada[pl] OR Guatemala[pl] OR Guinea[pl] OR Guam[pl] OR Guiana[pl] OR Guyana[pl] OR Haiti[pl] OR Honduras[pl] OR Hungary[pl] OR India[pl] OR Maldives[pl] OR Indonesia[pl] OR Iran[pl] OR Iraq[pl] OR Isle of Man[pl] OR Jamaica[pl] OR Jordan[pl] OR Kazakhstan[pl] OR Kazakh[pl] OR Kenya[pl] OR Kiribati[pl] OR Korea[pl] OR Kosovo[pl] OR Kyrgyzstan[pl] OR Kirghizia[pl] OR Kyrgyz Republic[pl] OR Kirghiz[pl] OR Kirgizstan[pl] OR "Lao PDR"[pl] OR Laos[pl] OR Latvia[pl] OR Lebanon[pl] OR Lesotho[pl] OR Basutoland[pl] OR Liberia[pl] OR Libya[pl] OR Lithuania[pl]

**#6 Search** Macedonia[pl] OR Madagascar[pl] OR Malagasy Republic[pl] OR Malaysia[pl] OR Malaya[pl] OR Malay[pl] OR Sabah[pl] OR Sarawak[pl] OR Malawi[pl] OR Nyasaland[pl] OR Mali[pl] OR Malta[pl] OR Marshall Islands[pl] OR Mauritania[pl] OR Mauritius[pl] OR Agalega Islands[pl] OR Mexico[pl] OR Micronesia[pl] OR Middle East[pl] OR Moldova[pl] OR Moldovia[pl] OR Moldovian[pl] OR Mongolia[pl] OR Montenegro[pl] OR Morocco[pl] OR Ifni[pl] OR Mozambique[pl] OR Myanmar[pl] OR Myanma[pl] OR Burma[pl] OR Namibia[pl] OR Nepal[pl] OR Netherlands Antilles[pl] OR New Caledonia[pl] OR Nicaragua[pl] OR Niger[pl] OR Nigeria[pl] OR Northern Mariana Islands[pl] OR Oman[pl] OR Muscat[pl] OR Pakistan[pl] OR Palau[pl] OR Palestine[pl] OR Panama[pl] OR Paraguay[pl] OR Peru[pl] OR Philippines[pl] OR Philipines[pl] OR Phillipines[pl] OR Phillippines[pl] OR Poland[pl] OR Portugal[pl] OR Puerto Rico[pl] OR Romania[pl] OR Rumania[pl] OR Roumania[pl] OR Russia[pl] OR Russian[pl] OR Rwanda[pl] OR Ruanda[pl] OR Saint Kitts[pl] OR St Kitts[pl] OR Nevis[pl] OR Saint Lucia[pl] OR St Lucia[pl] OR Saint Vincent[pl] OR St Vincent[pl] OR Grenadines[pl] OR Samoa[pl] OR Samoan Islands[pl] OR Navigator Island[pl] OR Navigator Islands[pl] OR Sao Tome[pl] OR Saudi Arabia[pl] OR Senegal[pl] OR Serbia[pl] OR Montenegro[pl] OR Seychelles[pl] OR Sierra Leone[pl] OR Slovenia[pl] OR Sri Lanka[pl] OR Ceylon[pl] OR Solomon Islands[pl] OR Somalia[pl] OR South Africa[pl] OR Sudan[pl] OR Suriname[pl] OR Surinam[pl] OR Swaziland[pl] OR Syria[pl] OR Tajikistan[pl] OR Tadzhikistan[pl] OR Tadjikistan[pl] OR Tadzhik[pl] OR Tanzania[pl] OR Thailand[pl] OR Togo[pl] OR Togolese Republic[pl] OR Tonga[pl] OR Trinidad[pl] OR Tobago[pl] OR Tunisia[pl] OR Turkey[pl] OR Turkmenistan[pl] OR Turkmen[pl] OR Uganda[pl] OR Ukraine[pl] OR Uruguay[pl] OR USSR[pl] OR Soviet Union[pl] OR Union of Soviet Socialist Republics[pl] OR Uzbekistan[pl] OR Uzbek OR Vanuatu[pl] OR New Hebrides[pl] OR Venezuela[pl] OR Vietnam[pl] OR Viet Nam[pl] OR West Bank[pl] OR Yemen[pl] OR Yugoslavia[pl] OR Zambia[pl] OR Zimbabwe[pl] OR Rhodesia[pl]

**#5 Search** Africa[tiab] OR Asia[tiab] OR Caribbean[tiab] OR West Indies[tiab] OR South America[tiab] OR Latin America[tiab] OR Central America[tiab] OR Afghanistan[tiab] OR Albania[tiab] OR Algeria[tiab] OR Angola[tiab] OR Antigua[tiab] OR Barbuda[tiab] OR Argentina[tiab] OR Armenia[tiab] OR Armenian[tiab] OR Aruba[tiab] OR Azerbaijan[tiab] OR Bahrain[tiab] OR Bangladesh[tiab] OR Barbados[tiab] OR Benin[tiab] OR Byelarus[tiab] OR Byelorussian[tiab] OR Belarus[tiab] OR Belorussian[tiab] OR Belorussia[tiab] OR Belize[tiab] OR Bhutan[tiab] OR Bolivia[tiab] OR Bosnia[tiab] OR Herzegovina[tiab] OR Hercegovina[tiab] OR Botswana[tiab] OR Brasil[tiab] OR Brazil[tiab] OR Bulgaria[tiab] OR Burkina Faso[tiab] OR Burkina Fasso[tiab] OR Upper Volta[tiab] OR Burundi[tiab] OR Urundi[tiab] OR Cambodia[tiab] OR Khmer Republic[tiab] OR Kampuchea[tiab] OR Cameroon[tiab] OR Cameroons[tiab] OR Cameron[tiab] OR Camerons[tiab] OR Cape Verde[tiab] OR Central African Republic[tiab] OR Chad[tiab] OR Chile[tiab] OR China[tiab] OR Colombia[tiab] OR Comoros[tiab] OR Comoro Islands[tiab] OR Comores[tiab] OR Mayotte[tiab] OR Congo[tiab] OR Zaire[tiab] OR Costa Rica[tiab] OR Cote d'Ivoire[tiab] OR Ivory Coast[tiab] OR Croatia[tiab] OR Cuba[tiab] OR Cyprus[tiab] OR Czechoslovakia[tiab] OR Czech Republic[tiab] OR Slovakia[tiab] OR Slovak Republic[tiab] OR Djibouti[tiab] OR French Somaliland[tiab] OR Dominica[tiab] OR Dominican Republic[tiab] OR East Timor[tiab] OR East Timur[tiab] OR Timor Leste[tiab] OR Ecuador[tiab] OR Egypt[tiab] OR United Arab Republic[tiab] OR El Salvador[tiab] OR Eritrea[tiab] OR Estonia[tiab] OR Ethiopia[tiab] OR Fiji[tiab] OR Gabon[tiab] OR Gabonese Republic[tiab] OR Gambia[tiab] OR Gaza[tiab] OR Georgia Republic[tiab] OR Georgian Republic[tiab] OR Ghana[tiab] OR Gold Coast[tiab] OR Greece[tiab] OR Grenada[tiab] OR Guatemala[tiab] OR Guinea[tiab] OR Guam[tiab] OR Guiana[tiab] OR Guyana[tiab] OR Haiti[tiab] OR Honduras[tiab] OR Hungary[tiab] OR India[tiab] OR Maldives[tiab] OR Indonesia[tiab] OR Iran[tiab] OR Iraq[tiab] OR Isle of Man[tiab] OR Jamaica[tiab] OR Jordan[tiab] OR Kazakhstan[tiab] OR Kazakh[tiab] OR Kenya[tiab] OR Kiribati[tiab] OR Korea[tiab] OR Kosovo[tiab] OR Kyrgyzstan[tiab] OR Kirghizia[tiab] OR Kyrgyz Republic[tiab] OR Kirghiz[tiab] OR Kirgizstan[tiab] OR "Lao PDR"[tiab] OR Laos[tiab] OR Latvia[tiab] OR Lebanon[tiab] OR Lesotho[tiab] OR Basutoland[tiab] OR Liberia[tiab] OR Libya[tiab] OR Lithuania[tiab]

**#4 Search** Macedonia[tiab] OR Madagascar[tiab] OR Malagasy Republic[tiab] OR Malaysia[tiab] OR Malaya[tiab] OR Malay[tiab] OR Sabah[tiab] OR Sarawak[tiab] OR Malawi[tiab] OR Nyasaland[tiab] OR Mali[tiab] OR Malta[tiab] OR Marshall Islands[tiab] OR Mauritania[tiab] OR Mauritius[tiab] OR Agalega Islands[tiab] OR Mexico[tiab] OR Micronesia[tiab] OR Middle East[tiab] OR Moldova[tiab] OR Moldovia[tiab] OR Moldovian[tiab] OR Mongolia[tiab] OR Montenegro[tiab] OR Morocco[tiab] OR Ifni[tiab] OR Mozambique[tiab] OR Myanmar[tiab] OR Myanma[tiab] OR Burma[tiab] OR Namibia[tiab] OR Nepal[tiab] OR Netherlands Antilles[tiab] OR New Caledonia[tiab] OR Nicaragua[tiab] OR Niger[tiab] OR Nigeria[tiab] OR Northern Mariana Islands[tiab] OR Oman[tiab] OR Muscat[tiab] OR Pakistan[tiab] OR Palau[tiab] OR Palestine[tiab] OR Panama[tiab] OR Paraguay[tiab] OR Peru[tiab] OR Philippines[tiab] OR Philipines[tiab] OR Phillipines[tiab] OR Phillippines[tiab] OR Poland[tiab] OR Portugal[tiab] OR Puerto Rico[tiab] OR Romania[tiab] OR Rumania[tiab] OR Roumania[tiab] OR Russia[tiab] OR Russian[tiab] OR Rwanda[tiab] OR Ruanda[tiab] OR Saint Kitts[tiab] OR St Kitts[tiab] OR Nevis[tiab] OR Saint Lucia[tiab] OR St Lucia[tiab] OR Saint Vincent[tiab] OR St Vincent[tiab] OR Grenadines[tiab] OR Samoa[tiab] OR Samoan Islands[tiab] OR Navigator Island[tiab] OR Navigator Islands[tiab] OR Sao Tome[tiab] OR Saudi Arabia[tiab] OR Senegal[tiab] OR Serbia[tiab] OR Montenegro[tiab] OR Seychelles[tiab] OR Sierra Leone[tiab] OR Slovenia[tiab] OR Sri Lanka[tiab] OR Ceylon[tiab] OR Solomon Islands[tiab] OR Somalia[tiab] OR Sudan[tiab] OR Suriname[tiab] OR Surinam[tiab] OR Swaziland[tiab] OR Syria[tiab] OR Tajikistan[tiab] OR Tadzhikistan[tiab] OR Tadjikistan[tiab] OR Tadzhik[tiab] OR Tanzania[tiab] OR Thailand[tiab] OR Togo[tiab] OR Togolese Republic[tiab] OR Tonga[tiab] OR Trinidad[tiab] OR Tobago[tiab] OR Tunisia[tiab] OR Turkey[tiab] OR Turkmenistan[tiab] OR Turkmen[tiab] OR Uganda[tiab] OR Ukraine[tiab] OR Uruguay[tiab] OR USSR[tiab] OR Soviet Union[tiab] OR Union of Soviet Socialist Republics[tiab] OR Uzbekistan[tiab] OR Uzbek OR Vanuatu[tiab] OR New Hebrides[tiab] OR Venezuela[tiab] OR Vietnam[tiab] OR Viet Nam[tiab] OR West Bank[tiab] OR Yemen[tiab] OR Yugoslavia[tiab] OR Zambia[tiab] OR Zimbabwe[tiab] OR Rhodesia[tiab]

**#3 Search** Africa[ot] OR Asia[ot] OR Caribbean[ot] OR West Indies[ot] OR South America[ot] OR Latin America[ot] OR Central America[ot] OR Afghanistan[ot] OR Albania[ot] OR Algeria[ot] OR Angola[ot] OR Antigua[ot] OR Barbuda[ot] OR Argentina[ot] OR Armenia[ot] OR Armenian[ot] OR Aruba[ot] OR Azerbaijan[ot] OR Bahrain[ot] OR Bangladesh[ot] OR Barbados[ot] OR Benin[ot] OR Byelarus[ot] OR Byelorussian[ot] OR Belarus[ot] OR Belorussian[ot] OR Belorussia[ot] OR Belize[ot] OR Bhutan[ot] OR Bolivia[ot] OR Bosnia[ot] OR Herzegovina[ot] OR Hercegovina[ot] OR Botswana[ot] OR Brasil[ot] OR Brazil[ot] OR Bulgaria[ot] OR Burkina Faso[ot] OR Burkina Fasso[ot] OR Upper Volta[ot] OR Burundi[ot] OR Urundi[ot] OR Cambodia[ot] OR Khmer Republic[ot] OR Kampuchea[ot] OR Cameroon[ot] OR Cameroons[ot] OR Cameron[ot] OR Camerons[ot] OR Cape Verde[ot] OR Central African Republic[ot] OR Chad[ot] OR Chile[ot] OR China[ot] OR Colombia[ot] OR Comoros[ot] OR Comoro Islands[ot] OR Comores[ot] OR Mayotte[ot] OR Congo[ot] OR Zaire[ot] OR Costa Rica[ot] OR Cote d'Ivoire[ot] OR Ivory Coast[ot] OR Croatia[ot] OR Cuba[ot] OR Cyprus[ot] OR Czechoslovakia[ot] OR Czech Republic[ot] OR Slovakia[ot] OR Slovak Republic[ot] OR Djibouti[ot] OR French Somaliland[ot] OR Dominica[ot] OR Dominican Republic[ot] OR East Timor[ot] OR East Timur[ot] OR Timor Leste[ot] OR Ecuador[ot] OR Egypt[ot] OR United Arab Republic[ot] OR El Salvador[ot] OR Eritrea[ot] OR Estonia[ot] OR Ethiopia[ot] OR Fiji[ot] OR Gabon[ot] OR Gabonese Republic[ot] OR Gambia[ot] OR Gaza[ot] OR "Georgia Republic"[ot] OR "Georgian Republic"[ot] OR Ghana[ot] OR Gold Coast[ot] OR Greece[ot] OR Grenada[ot] OR Guatemala[ot] OR Guinea[ot] OR Guam[ot] OR Guiana[ot] OR Guyana[ot] OR Haiti[ot] OR Honduras[ot] OR Hungary[ot] OR India[ot] OR Maldives[ot] OR Indonesia[ot] OR Iran[ot] OR Iraq[ot] OR Isle of Man[ot] OR Jamaica[ot] OR Jordan[ot] OR Kazakhstan[ot] OR Kazakh[ot] OR Kenya[ot] OR Kiribati[ot] OR Korea[ot] OR Kosovo[ot] OR Kyrgyzstan[ot] OR Kirghizia[ot] OR Kyrgyz Republic[ot] OR Kirghiz[ot] OR Kirgizstan[ot] OR "Lao PDR"[ot] OR Laos[ot] OR Latvia[ot] OR Lebanon[ot] OR Lesotho[ot] OR Basutoland[ot] OR Liberia[ot] OR Libya[ot] OR Lithuania[ot]

**#2 Search** Macedonia[ot] OR Madagascar[ot] OR Malagasy Republic[ot] OR Malaysia[ot] OR Malaya[ot] OR Malay[ot] OR Sabah[ot] OR Sarawak[ot] OR Malawi[ot] OR Nyasaland[ot] OR Mali[ot] OR Malta[ot] OR Marshall Islands[ot] OR Mauritania[ot] OR Mauritius[ot] OR Agalega Islands[ot] OR Mexico[ot] OR Micronesia[ot] OR Middle East[ot] OR Moldova[ot] OR Moldovia[ot] OR Moldovian[ot] OR Mongolia[ot] OR Montenegro[ot] OR Morocco[ot] OR Ifni[ot] OR Mozambique[ot] OR Myanmar[ot] OR Myanma[ot] OR Burma[ot] OR Namibia[ot] OR Nepal[ot] OR Netherlands Antilles[ot] OR New Caledonia[ot] OR Nicaragua[ot] OR Niger[ot] OR Nigeria[ot] OR Northern Mariana Islands[ot] OR Oman[ot] OR Muscat[ot] OR Pakistan[ot] OR Palau[ot] OR Palestine[ot] OR Panama[ot] OR Paraguay[ot] OR Peru[ot] OR Philippines[ot] OR Philipines[ot] OR Phillipines[ot] OR Phillippines[ot] OR Poland[ot] OR Portugal[ot] OR Puerto Rico[ot] OR Romania[ot] OR Rumania[ot] OR Roumania[ot] OR Russia[ot] OR Russian[ot] OR Rwanda[ot] OR Ruanda[ot] OR Saint Kitts[ot] OR St Kitts[ot] OR Nevis[ot] OR Saint Lucia[ot] OR St Lucia[ot] OR Saint Vincent[ot] OR St Vincent[ot] OR Grenadines[ot] OR Samoa[ot] OR Samoan Islands[ot] OR Navigator Island[ot] OR Navigator Islands[ot] OR Sao Tome[ot] OR Saudi Arabia[ot] OR Senegal[ot] OR Serbia[ot] OR Montenegro[ot] OR Seychelles[ot] OR Sierra Leone[ot] OR Slovenia[ot] OR Sri Lanka[ot] OR Ceylon[ot] OR Solomon Islands[ot] OR Somalia[ot] OR Sudan[ot] OR Suriname[ot] OR Surinam[ot] OR Swaziland[ot] OR Syria[ot] OR Tajikistan[ot] OR Tadzhikistan[ot] OR Tadjikistan[ot] OR Tadzhik[ot] OR Tanzania[ot] OR Thailand[ot] OR Togo[ot] OR Togolese Republic[ot] OR Tonga[ot] OR Trinidad[ot] OR Tobago[ot] OR Tunisia[ot] OR Turkey[ot] OR Turkmenistan[ot] OR Turkmen[ot] OR Uganda[ot] OR Ukraine[ot] OR Uruguay[ot] OR USSR[ot] OR Soviet Union[ot] OR Union of Soviet Socialist Republics[ot] OR Uzbekistan[ot] OR Uzbek OR Vanuatu[ot] OR New Hebrides[ot] OR Venezuela[ot] OR Vietnam[ot] OR Viet Nam[ot] OR West Bank[ot] OR Yemen[ot] OR Yugoslavia[ot] OR Zambia[ot] OR Zimbabwe[ot] OR Rhodesia[ot]

**#1 Search** Developing Countries[Mesh:noexp] OR Africa[Mesh:noexp] OR Africa, Northern[Mesh:noexp] OR Africa South of the Sahara[Mesh:noexp] OR Africa, Central[Mesh:noexp] OR Africa, Eastern[Mesh:noexp] OR Africa, Southern[Mesh:noexp] OR Africa, Western[Mesh:noexp] OR Asia[Mesh:noexp] OR Asia, Central[Mesh:noexp] OR Asia, Southeastern[Mesh:noexp] OR Asia, Western[Mesh:noexp] OR Caribbean Region[Mesh:noexp] OR West Indies[Mesh:noexp] OR South America[Mesh:noexp] OR Latin America[Mesh:noexp] OR Central America[Mesh:noexp] OR Afghanistan[Mesh:noexp] OR Albania[Mesh:noexp] OR Algeria[Mesh:noexp] OR American Samoa[Mesh:noexp] OR Angola[Mesh:noexp] OR "Antigua and Barbuda"[Mesh:noexp] OR Argentina[Mesh:noexp] OR Armenia[Mesh:noexp] OR Azerbaijan[Mesh:noexp] OR Bahrain[Mesh:noexp] OR Bangladesh[Mesh:noexp] OR Barbados[Mesh:noexp] OR Benin[Mesh:noexp] OR Byelarus[Mesh:noexp] OR Belize[Mesh:noexp] OR Bhutan[Mesh:noexp] OR Bolivia[Mesh:noexp] OR Bosnia-Herzegovina[Mesh:noexp] OR Botswana[Mesh:noexp] OR Brazil[Mesh:noexp] OR Bulgaria[Mesh:noexp] OR Burkina Faso[Mesh:noexp] OR Burundi[Mesh:noexp] OR Cambodia[Mesh:noexp] OR Cameroon[Mesh:noexp] OR Cape Verde[Mesh:noexp] OR Central African Republic[Mesh:noexp] OR Chad[Mesh:noexp] OR Chile[Mesh:noexp] OR China[Mesh:noexp] OR Colombia[Mesh:noexp] OR Comoros[Mesh:noexp] OR Congo[Mesh:noexp] OR Costa Rica[Mesh:noexp] OR Cote d'Ivoire[Mesh:noexp] OR Croatia[Mesh:noexp] OR Cuba[Mesh:noexp] OR Cyprus[Mesh:noexp] OR Czechoslovakia[Mesh:noexp] OR Czech Republic[Mesh:noexp] OR Slovakia[Mesh:noexp] OR Djibouti[Mesh:noexp] OR "Democratic Republic of the Congo"[Mesh:noexp] OR Dominica[Mesh:noexp] OR Dominican Republic[Mesh:noexp] OR East Timor[Mesh:noexp] OR Ecuador[Mesh:noexp] OR Egypt[Mesh:noexp] OR El Salvador[Mesh:noexp] OR Eritrea[Mesh:noexp] OR Estonia[Mesh:noexp] OR Ethiopia[Mesh:noexp] OR Fiji[Mesh:noexp] OR Gabon[Mesh:noexp] OR Gambia[Mesh:noexp] OR "Georgia (Republic)"[Mesh:noexp] OR Ghana[Mesh:noexp] OR Greece[Mesh:noexp] OR Grenada[Mesh:noexp] OR Guatemala[Mesh:noexp] OR Guinea[Mesh:noexp] OR Guinea-Bissau[Mesh:noexp] OR Guam[Mesh:noexp] OR Guyana[Mesh:noexp] OR Haiti[Mesh:noexp] OR Honduras[Mesh:noexp] OR Hungary[Mesh:noexp] OR India[Mesh:noexp] OR Indonesia[Mesh:noexp] OR Iran[Mesh:noexp] OR Iraq[Mesh:noexp] OR Jamaica[Mesh:noexp] OR Jordan[Mesh:noexp] OR Kazakhstan[Mesh:noexp] OR Kenya[Mesh:noexp] OR Korea[Mesh:noexp] OR Kosovo[Mesh:noexp] OR Kyrgyzstan[Mesh:noexp] OR Laos[Mesh:noexp] OR Latvia[Mesh:noexp] OR Lebanon[Mesh:noexp] OR Lesotho[Mesh:noexp] OR Liberia[Mesh:noexp] OR Libya[Mesh:noexp] OR Lithuania[Mesh:noexp] OR Macedonia[Mesh:noexp] OR Madagascar[Mesh:noexp] OR Malaysia[Mesh:noexp] OR Malawi[Mesh:noexp] OR Mali[Mesh:noexp] OR Malta[Mesh:noexp] OR Mauritania[Mesh:noexp] OR Mauritius[Mesh:noexp] OR Mexico[Mesh:noexp] OR Micronesia[Mesh:noexp] OR Middle East[Mesh:noexp] OR Moldova[Mesh:noexp] OR Mongolia[Mesh:noexp] OR Montenegro[Mesh:noexp] OR Morocco[Mesh:noexp] OR Mozambique[Mesh:noexp] OR Myanmar[Mesh:noexp] OR Namibia[Mesh:noexp] OR Nepal[Mesh:noexp] OR Netherlands Antilles[Mesh:noexp] OR New Caledonia[Mesh:noexp] OR Nicaragua[Mesh:noexp] OR Niger[Mesh:noexp] OR Nigeria[Mesh:noexp] OR Oman[Mesh:noexp] OR Pakistan[Mesh:noexp] OR Palau[Mesh:noexp] OR Panama[Mesh:noexp] OR Papua New Guinea[Mesh:noexp] OR Paraguay[Mesh:noexp] OR Peru[Mesh:noexp] OR Philippines[Mesh:noexp] OR Poland[Mesh:noexp] OR Portugal[Mesh:noexp] OR Puerto Rico[Mesh:noexp] OR Romania[Mesh:noexp] OR Russia[Mesh:noexp] OR "Russia (Pre-1917)"[Mesh:noexp] OR Rwanda[Mesh:noexp] OR "Saint Kitts and Nevis"[Mesh:noexp] OR Saint Lucia[Mesh:noexp] OR "Saint Vincent and the Grenadines"[Mesh:noexp] OR Samoa[Mesh:noexp] OR Saudi Arabia[Mesh:noexp] OR Senegal[Mesh:noexp] OR Serbia[Mesh:noexp] OR Montenegro[Mesh:noexp] OR Seychelles[Mesh:noexp] OR Sierra Leone[Mesh:noexp] OR Slovenia[Mesh:noexp] OR Sri Lanka[Mesh:noexp] OR Somalia[Mesh:noexp] OR South Africa[Mesh:noexp] OR Sudan[Mesh:noexp] OR Suriname[Mesh:noexp] OR Swaziland[Mesh:noexp] OR Syria[Mesh:noexp] OR Tajikistan[Mesh:noexp] OR Tanzania[Mesh:noexp] OR Thailand[Mesh:noexp] OR Togo[Mesh:noexp] OR Tonga[Mesh:noexp] OR "Trinidad and Tobago"[Mesh:noexp] OR Tunisia[Mesh:noexp] OR Turkey[Mesh:noexp] OR Turkmenistan[Mesh:noexp] OR Uganda[Mesh:noexp] OR Ukraine[Mesh:noexp] OR Uruguay[Mesh:noexp] OR USSR[Mesh:noexp] OR Uzbekistan[Mesh:noexp] OR Vanuatu[Mesh:noexp] OR Venezuela[Mesh:noexp] OR Vietnam[Mesh:noexp] OR Yemen[Mesh:noexp] OR Yugoslavia[Mesh:noexp] OR Zambia[Mesh:noexp] OR Zimbabwe[Mesh:noexp]

**Appendix B: Data extraction sheet (fragmented Excel)**
