# Supplementary material for: Strategies for intrapartum foetal surveillance in low- and middle-income countries: A systematic review
Source: PLoS One. 2018 Oct 26;13(10):e0206295. doi: 10.1371/journal.pone.0206295 (PMC6203373; doi:10.1371/journal.pone.0206295)
Supplement: S1 Table — (DOCX) [file pone.0206295.s006.docx]

| **Strategy of foetal surveillance** | **Major quantitative results** |
| --- | --- |
| **Admission tests:**  **- CTG** | - Low-risk pregnancies: correlation between reactive admission test and good neonatal outcome (diagnostic accuracy of 93.75%), equivocal and ominous traces are associated with Apgar score <7 (4.87% compared to ominous 68.4%, p=0.0001) and NICU admission (1.1% compared to ominous 47.4%, p=0.03), CS (30.0% compared to ominous 100%, p<0.0001).[40,43,55]  - High-risk pregnancies: worsening CTG associated with foetal distress (reactive: 11%, ominous: 86%); Apgar score <7 at 5 minutes (reactive:6.5%, ominous:57.1%); cord blood pH <7.2(reactive: 4.1%, ominous: 57.1), NICU admission: (reactive: 6.5%, ominous: 57.1%.); p-values<0.02.[39,46,59]  - Mixed-risk pregnancies: low sensitivity (40%) but high specificity (92%) for foetal compromise.[44]  - Worsening CTG associated with mode of delivery. Low-risk pregnancies: Vaginal: Reactive: 67.0%, Ominous: 0%, p<0.0001; Caesarean section: Reactive: 30.0%, Ominous: 100%, p<0.05. High-risk pregnancies: CS: Reactive: 35.8%, Ominous:[40,44,46,55]  - Non-reactive NST: significantly more stillbirths (Non-reactive NST: 2.7%, Reactive NST: 0%, p<0.001), low Apgar scores (Non-reactive NST: 19.2%, Reactive NST: 0%, p<0.001).[40,43,44,55,59] |
| **- Intermittent Auscultation** | - Hand-held Doppler can accurately determine foetal viability on admission: Stillbirths: FHR present: 0.55%, FHR absent: 89.2%; Alive at discharge: FHR present: 98.69%, FHR absent: 6.15%.[42] |
| **- Amniotic Fluid Index** | - Decreased AFI to be significant for NICU admission in high risk group (<5cm:75%, 8.1-20cm: 7.0%) and CS (<5cm 83.3%, 8.1-20cm: 6.1%). [47]  - Low test performance (sensitivity 58%, specificity 64%, positive predictive value 24%, negative predictive value 88%).[47] |
| **Intermittent Auscultation**  **Doppler /Pinard** | - Abnormal FHR is associated with fresh stillbirth (0.3% to 47.5%, p<0.0005), need for resuscitation (OR: 7.8 (95% CI: 5.9-10.1), p<0.0005), Apgar score <7 at 5 minutes (OR: 21.7 (95% CI: 12.7-37.0), p<0.0005), early neonatal death (OR: 9.9 (95% CI: 5.6-17.5), p<0.0005), NICU admission (OR: 3.0 (95% CI: 1.2–7.8), p=0.023).[48]  - Pinard and Hand-held Doppler: Doppler detected more FHR abnormalities (4.7% to 7.6%, p<0.05); no differences on neonatal outcomes (neonatal death 0.5% to 0.7%, p=0.58, Apgar scores 1.7% to 2.3%, p=0.40, NICU admission (3.7% to 4.8%, p=0.20) or number of CS (17.0% to 17.6%, p=0.695).[30–32]  - Foetal distress diagnosed on the combination of FHR abnormalities and meconium: predicts low 1 and 5 minute Apgar scores (59.0% to 31.0%, p=0.003, 24.3% to 3.4% p=0.001), and NICU admission (31% to 17%).[51,53,54,57]  - CTG to IA: No difference in maternal morbidity (postpartum haemorrhage p=0.494 and infection p=1.0).[32] |
|  |  |
| **Electronic Foetal Monitoring**  **External/Internal** | - Pathological and suspected pathological CTGs correlated with adverse neonatal outcomes: 1 minute Apgar scores ≤4 (29.2% and 90.4%, p=0.002) and low 5 minute Apgar scores (8.3% and 3.8%, p<0.42). [34,51]  - Foetal heart rate abnormalities did not correlate with adverse neonatal outcomes: 5 minute Apgar score <7 (15.2%), cord pH <7.10 (5.9%) and NICU admission (15.2%). [34,49,54,61]  - Test performance: suspicious CTG has low predictive value for foetal acid-base state and pathological CTG has high predictive value for foetal acid-base state (13%-35% and 50-100% respectively). [34,49,54,61]  - Presence of FHR accelerations correlates with significantly less lower Apgar scores (15% to 5%).  - Cases of HIE: significant higher percentage of pathological CTG findings in cases, compared in the neonates without HIE (66.2% to 27.5%, p<0.05). [37]  **-** CTG to IA: Detection of abnormal foetal heart rate: CTG (54%), hand-held Doppler (32%) and Pinard (9%-15%) (p<0.05).[30] IA detects less abnormal FHR than CTG (P<0.000) [30–32]  - CTG to IA: non-significant for low 1 min and 5 Apgar (6% to 8%, p=1.0, 2% to 6%, p=0.62), NICU admission (2% to 8%, p=0.36), vaginal delivery (64% to 72%, p=0.39), CS (34% to 22%, p=0.18).[32] No difference in maternal morbidity and neonatal outcome (p>0.05).(30–32) |
|  |  |
| **Meconium** | **-** Meconium poor indicator of perinatal outcomes but combination of abnormal FHR and meconium liquor: increased the odds of severe neonatal compromise at birth (OR:3.84 (CL 1.90-7.77), p=0.0002).  - Thick meconium: increase in low Apgar scores at 1 minute (5.3% to 50%), NICU admissions (clear 2.9% to 11.1%) and an insignificant increase in instrumental vaginal delivery (17.8% to 27.7%).  - High specificity and low sensitivity to predict umbilical cord arterial base excess (> 12mmol/L) (79.2% and 18.8% respectively). [51,53,54,57] |
| **Foetal Scalp Stimulation Test** | **-** Non-reactivity to the FSST associated with adverse neonatal outcomes: a significantly lower umbilical cord blood pH (pH<7.0: Reactive FSST: 2.2%, Non-reactive FSST: 12.6%), lower Apgar scores at 1 and 5 minutes (Reactive FSST: 0%, Non-reactive FSST: 2.3%) and a higher incidence of asphyxia related neonatal death and morbidity (Reactive FSST: 0.7%, Non-reactive FSST: 5.3%). [56] |
| **Foetal Acoustic Stimulation Test** | **-** Increases test performance of NST in predicting 5 minute Apgar score significantly (NST before FAST and after FAST: Sensitivity: 100%, 100%; Specificity: 63.3%, 96.9%; PPV: 19.2%, 73.6%; NPV: 100%, 100%).  - Maternal perception of sound-provoked foetal movement was of poor perinatal outcomes (thick meconium, stained amniotic fluid, perinatal death, Apgar score <7 at 5minutes, admission to the NICU and foetal distress), sensitivity: l00%, specificity: 98.9%, PPV: 15.1%, NPV:100%, accuracy: 90.1%. [39,43] |
| **Foetal Pulse Oximetry &Foetal Blood Sampling** | - A FPO30 cut-off has diagnostic value similar to that of scalp blood analysis in terms of umbilical cord pH (FPO30 and scalp blood: Sensitivity: 75%, 72%; Specificity: 49%, 53%) and adverse neonatal outcomes (FPO30 and scalp blood: Sensitivity: 89%, 82%; Specificity: 49%, 52%).[36] |
| **Rapid biophysical profile** | **-** rBPP has higher test performance for neonatal outcomes as compared to either AFI or sound provoked foetal movement on ultrasound alone (rBPP/AFI/SPFM: sensitivity: 50.0%, 33.3%, 33.3; Specificity: 99.1%, 89.8%, 95.7%; PPV: 50%, 5.7%, 12.5%, NPV: 99.1%, 98.6% and 98.6% respectively).[62] |
| **Umbilical artery Doppler velocimetry** | **-** No significance for umbilical artery resistance in the prediction of foetal compromise (zero change in mean Porcelot ratio in both acidotic/normal foetuses; Sensitivity 13%, Specificity 89%, PPV 25%, NPV 70%).[45,60] |
| **Partograph** | -WHO partograph: reduction in intrapartum stillbirth (0.50% to 0.31%, p=0.024) but not for vaginal breech delivery, reduced prolong labours (6.4% to 3.4%, p=0.002), reduced labour augmentation (20.7% to 9.1%, p=0.023), reduced postpartum sepsis (0.70% to 0.21, p=0.028). No significant reduction in neonatal morbidity, caesarean section and maternal mortality.[27,29]  - Crossing the alert line is associated with the need for neonatal resuscitation (p≤0.001) and fresh stillbirths (p<0.01). [38,63]  - Training midwives to use the partogram: reduced poor Apgar at 1 minute (26.1% to 14.8%, p=0.001), but no significant reduction in poor Apgar at 5 minutes (7.8% to 6.6% p=0.402), stillbirths (2.2% to1.6%, p**=**0.613), neonatal deaths (2.2% to 1.0%, p=0.193) and need for resuscitation (5.0% to 3.6%, p = 0.222).[33] Substandard use of partograph was associated with low Apgar score.[52] |

**Abbreviations:** CTG = Cardiotocograph, CS = Caesarean Section, FHR = Foetal Heart Rate, AFI = Amniotic Fluid Index, NICU = Neonatal Intensive Care Unit, IA = Intermittent Auscultation, FSST = Foetal Scalp Stimulation Test, FAST = Foetal Acoustic stimulation Test, FPO = Foetal Pulse Oximetry, rBPP = rapid Biophysical Profile, SPFM = Sound-Provoked Foetal Movement, NST = Non-Stress Test, CI = Confidence Interval, OR = Odds Ratio, CI = Confidence Interval, PPV = Positive Predictive Value, NPV = Negative Predictive Value, HIE = Hypoxic Ischaemic Encephalopathy, EFM = Electronic Foetal Monitoring, S = Strength, W = Weakness, O = Opportunity, T= Threat (SWOT), WHO = World Health Organisation
