# Supplementary material for: Strategies for intrapartum foetal surveillance in low- and middle-income countries: A systematic review
Source: PLoS One. 2018 Oct 26;13(10):e0206295. doi: 10.1371/journal.pone.0206295 (PMC6203373; doi:10.1371/journal.pone.0206295)
Supplement: S2 Table — (DOCX) [file pone.0206295.s007.docx]

**S2 Table. Characteristics and results of the randomized controlled trials (n=5).**

| **Study** | **Strategy of foetal surveillance** | **Study Characteristics** | **Facility characteristics** | **Strategy of implementation** | **Study population** | **Neonatal and Maternal Outcomes** |  | **Test**  **performance** |
| --- | --- | --- | --- | --- | --- | --- | --- | --- |
| Byaruhanga et al. 2015 | **Intervention**: Doppler  N=992.  **Control:** Pinard stethoscope  N=979. | RCT. Uganda (LIC). | Peri-urban, private, non-profit, teaching hospital. Annual deliveries: 7500. CTG unavailable. | Pre-study and in-service training: All midwives and doctors were given half a day of training (Helping Babies Survive Labour), FHR monitoring: every 30 mins in first stage, every 15 mins in second stage before pushing, every 5 mins when pushing, 1 min after contraction. Partograph was used. | N=1971, singleton, cephalic, at term. | **INTRAPARUM STILLBIRTH: Pinard:** 0.1% (1/979), **Doppler** 0.4% (4/992), IRR:3.94 (95% CI 0.44-35.24), p=0.184  **NEONATAL DEATH BEFORE DISCHARGE: Pinard**: 0.5% (5/979), **Doppler** 0.7% (7/992), p=0.58  **APGAR SCORE <7 AT 5 MIN:** **Pinard:** 1.7% (17/979), **Doppler:** 2.3% (23/992), p=0.40  **NICU ADMISSION:** **Pinard:** 3.7% (36/979), **Doppler:** 4.8 (48/992), p=0.20  **ABNORMAL FHR: Pinard:** 4.7% (46/979), **Doppler:** 7.6% (75/992), IRR: 1.61, (95% CI 1.13-2.30), p=0.008  **CS:** **Pinard:** 17.0%(166/979), **Doppler:** 17.6% (175/992), p=0.695 | | **NA** |
| Fahdhy et al. 2005 | **Intervention:**  Partograph (modified WHO): N=304 in trained midwife arm.  **Control:**  Standard midwifery care without use of partographs: N=322 pregnant woman under the care of 10 untrained midwifes. | RCT. Indonesia (LMIC). | Urban and rural, birthing homes. | A 2-day training programme on the use of the WHO partograph for 10 midwives for the intervention group (theory and practical). Referral to hospital at/after alert line  Supervision and monitoring of the project was done weekly for first month. | N=625, low-risk pregnancies. | **STILLBIRTH: Intervention:** 1.6% (5/304), **Control** 2.2% (7/322), Crude OR: 0.75 (0.25-2.28), p**=**0.613, Adjusted OR: 0.62 (0.17-2.19), p**=**0.456  **EARLY NEONATAL DEATH: Intervention:** 1.0% (3/304), **Control:** 2.2% (7/322), Crude OR: 0.45 (0.13-1.50), p=0.193, Adjusted OR: 0.70 (0.16-3.11), p=0.641  **APGAR <7 AT 1 MIN: Intervention:** 14.8% (45/304), **Control:** 26.1% (84/322), Crude OR: 0.49 (0.37-0.65), p=0.001, Adjusted OR: 0.45 (0.26-0.79), p**=**0.006  **APGAR <7 AT 5 MIN: Intervention:** 6.6% (20/304), **Control:** 7.8% (25/322), Crude OR: 0.83 (0.54-1.28), p=0.402, Adjusted OR: 1.01 (0.44-2.25), p=0.974  **CS: Intervention:** 4.9%(15/304), **Control:** 7.5%(24/322), Crude OR (0.45- 0.90), p=0.011 Adjusted OR: 0.49 (0.21-1.17), p=0.110  **RESUSCITATION: Intervention:** 3.6% (11/304), **Control:** 5.0% (16/322), Crude OR: 0.72 (0.43-1.22), p = 0.222, Adjusted OR: 0.92 (0.38-2.29), p = 0.855 | | **NA** |
| Madaan et al. 2006 | **Intervention:**  Intermittent auscultation.  **Contro**l:  CTG. | RCT. India (LMIC). | **NA** | Continuous CTG.  Intermittent auscultation: every 15 mins in first stage, every 5 mins in second stage, for 1 min after contraction. | N=100 post-caesarean pregnancies: singleton (n=50/n=50). | **ABNORMAL FHR: EFM**: 48% (24/50), **IA**: 10% (5/50), p<0.000028  **MECONIUM: EFM**: 22% (11/50), **IA**: 38% (19/50), p<0.080  **APGAR <7 AT 1 MIN:** **EFM:** 6% (3/50), **IA:** 8% (4/50), p=1.0  **APGAR <7 AT 5 MIN:** **EFM:** 2% (1/50), **IA:** 6% (3/50), p=0.62  **NICU ADMISSION:** **EFM:** 2% (1/50), **IA:** 8% (4/50), p=0.36  **VAGINAL DELIVERY:** **EFM:** 64% (32/50), **IA:** 72% (36/50), p=0.39  **FORCEPS:** **EFM:** 2% (1/50), **IA:** 6% (3/50), p=0.62  **CS:** **EFM:** 34% (17/50), **IA:** 22% (11/50), p=0.18  **MATERNAL OUTCOMES:** PPH, infection/fever, ruptured uterus: not significant | | **NA** |
| Mahomed et al. 1994 | **Intervention:**  CTG, Doppler, and Pinard stethoscope by a research midwife.  **Control:**  Pinard stethoscope by an attending midwife (routine care). | RCT. Zimbabwe (LIC). | Teaching, referral hospital. Annual delivery rate: 18.000 deliveries. No access to: foetal blood sampling or epidural analgesia. | Training and protocol provided before study commenced. Direct Supervision of intervention groups by research midwives at all times  CTG: external, interval CTG, continuous for 10mins every 30mins if normal or every 20mins if abnormal trace, assessment of decelerations and not of baseline variability  Doppler: last 10mins of every 30mins, before and after contraction  Pinard stethoscope: last 10mins of every 30mins, during and after contraction  Routine: Pinard stethoscope | N=1255 total, N=318 Intermittent CTG, N=312 Doppler, N=310 Pinard, N=315 Routine monitoring.  Cephalic pregnancies. | **INTRAPARTUM PERINATAL DEATHS: EFM:** 3% (8/318), **Doppler**: 0.6% (2/312), **Pinard:** 2% (5/310), **Routine:** 3% (9/315)  **APGAR <6 AT 5 MIN: EFM:** 2% (6/318), RR: 0.7 (0.2-1.8) **Doppler**: 1% (3/312), RR: 0.3 (0.1-1.2), **Pinard:** 3% (8/310), RR: 0.9 (0.4-2.3) **Routine:** 3% (9/315), p=0.40  **NICU ADMISSION: EFM**: 16% (51/318), **Doppler**: 11% (34/312), **Pinard:** 15% (47/310), **Routine:** 18% (57/315), p(Doppler vs. routine)=0.20  **FITS: EFM:** 0% (0/318), **Doppler:** 0% (0/312), **Pinard:** 2% (6/310), **Routine:** 3% (9/315)  **HIE: EFM:** 0.6% (2/318), **Doppler:** 0.3% (1/312), **Pinard:** 2% (7/310), **Routine:** 3% (10/315)  **CS: EFM** 28% (89/318), **Doppler** 24% (76/312), **Pinard:** 10%, (32/310), **Routine:** 15% (46/315), **RR**: 1.8 (1.4-2.3), 1.6 (1.2-2.0), 0.9 (0.6-1.2) compared to routine care respectively. **Due to expected Foetal distress**: 63%, 67%, 41%, 41%.  **OPERATIVE VAGINAL:** **EFM:** 10% (31/318), **Doppler:** 9% (28/312), **Pinard:** 8% (25/310), **Routine:** 7% (21/315)  **ABNORMAL FHR: EFM:** 54% (172/318)**,** RR: 6.1 (4.2-8.8), **Doppler:** 32% (100/312), RR: 3.6 (2.4-5.3) **Pinard:** 15% (47/310), RR: 1.7 (1.1-2.7), **Routine:** 9% (28/315)**, Prolonged early and late decelerations: EFM:** 21% (66/318), **Doppler:** 20% (62/312), **Pinard:** 10% (30/310), **Routine:** 7% (23/315) | | **NA** |
| WHO, 1994 | WHO Partograph. | Cluster RTC.  Thailand, Malaysia and Indonesia (UMIC).  Thailand, Malaysia and Indonesia (UMIC). | Four pairs of district general hospitals in Southeast Asia | After 5 months, the old WHO partograph was taught /introduced into randomly selected hospitals. After 10 months, it was introduced into the remaining hospitals and the study continued for 5 months. | N=35484, Before intervention: n=18254, After Intervention: n=17230 | **TOTAL STILLBIRTHS: ALL WOMEN: Before implementation:** 2.8%(516/18254) **After implementation:** 2.4% (413/17230), p=0.06.  **INTRAPARTUM STILLBIRTHS:** **ALL WOMEN: Before implementation:** 0.5%(93/18254,) **After implementation**: 0.3%(55/17230), p=0.024.  **NEONATAL DEATH:** **Before intervention:** 0.5%(89/18254), **After intervention:** 0.3%(50/17230), p=0.68.  **VAGINAL:**  **ALL WOMEN: SINGLETON PREGNANCIES: Before implementation:**72.4%(13186/18254), after implementation: 73.9%(12704/17230) p=0.201  **VACUUM/FORCEPS: ALL WOMEN: SINGLETON PREGNANCIES:** Before implementation: 9.8%(1793/18254), After implementation: 9.6%(1649/17230), p=0.110  **CS: (emergency): ALL WOMEN: SINGLETON PREGNANCIES: Before implementation:** 9.9%(1802/18254), **After implementation:** 8.7%(1495/17230), p=0.678. **MULTIPLE PREGNANCIES: ALL WOMEN: Before implementation:** 0.23%(41/18254), **After implantation** 0.22(37/17230), p=0.848  **DURATION OF LABOUR (median**): **ALL WOMEN:** **Before/after implementation:** 3.25hours/3.13, p=0.819, **Labour>18hours:** 6.4%(1147/18045)/3.4%(589/17132), p=0.002  **LABOUR AUGMENTED:** **ALL WOMEN: Before/After implementation**: 20.7%(3785/18254)/9.1%(1573/17230), p=0.023  **POSTPARTUM SEPSIS:** **ALL WOMEN: Before/After implementation**: 0.7%(127/18254)/0.21%(32/17230), p=0.028  **APGAR SCORE, NEONATAL RESUSCITATION, ADMISSION TO NICU:** No significant changes  **MATERNAL MORTALITY, POSTPARTUM HAEMORRHAGE:** No significant changes | |  |
| Lennox et.,1998 | WHO Partograph. | Cluster RTC.  Thailand, Malaysia and Indonesia (UMIC). | Four pairs of district general hospitals. | After 5 months, the old WHO partograph was taught /introduced into randomly selected hospitals. After 10 months, it was introduced into the remaining hospitals and the study continued for 5 months. | N=1740. Breech presentations. | **INTRAPARTUM STILLBIRTH: ALL WOMAN: Before implementation:** 1.9% (16/923), **After implementation:** 1.1% (8/817), **PRIMI: Before implementation**: 1.1% (4/379), **After implementation:** 0.6% (2/333). **MULTI: Before implementation:** 2.2% (12/542), **After implementation**: 1.2% (6/482).  **CS: ALL WOMAN: Before implementation:** 31.9% (294/923), **After implementation:** 27.3% (223/817), **PRIMI: Before implementation:** 38.5% (146/379), **After implementation:** 38.7% (129/333), **MULTI: Before implementation:** 27.1% (147/542), **After implementation:** 19.3% (93/482).  **NICU ADMISSION: ALL WOMAN: Before implementation:** 28.9% (239/923), **After implementation:** 22.5% (169/817), **PRIMI: Before implementation**: 30.6% (107/379), **After implementation:** 20.1% (63/333). **MULTI: Before implementation:** 27.7% (132/542), **After implementation**: 24.3% (106/482).  **APGAR SCORE AT 1: ALL WOMEN: Before implementation:** vaginal: 0-3: 8.2% (44/617), 4-7: 34.1% (184/617), 8-10: 57.7% (311/617), CS: 0-3: 4.5% (13/294), 4-7: 23.5% (68/294), 8-10: 72%(208/294), **After implementation:** vaginal: 0-3: 7.1% (38/589), 4-7: 38.8% (207/589), 8-10: 57.7% (288/589), CS: 0-3: 2.3% (5/223), 4-7: 33% (72/223), 8-10: 64% (141/233), **PRIMI:** **Before implementation:** vaginal: 0-3: 7.3% (15/229), 4-7: 37.9% (78/229), 8-10: 54.9% (113/229), CS: 0-3: 4.2% (6/146), 4-7: 22.2% (32/146), 8-10: 73.6% (106/146), **After implementation:** vaginal: 0-3: 9.7% (18/204), 4-7: 39.8% (74/204), 8-10: 50.5% (94/204), CS: 0-3: 2.4% (3/129), 4-7: 33.1% (42/129), 8-10: 64.6% (182/129), **MULTI: Before implementation:** vaginal: 0-3: 8.7% (29/387), 4-7: 31.9% (106/387), 8-10: 59.4% (252/387), CS: 0-3: 4.9% (7/147), 4-7: 24.3% (35/147), 8-10: 70.8% (105/147), **After implementation:** vaginal: 0-3: 5.8% (20/384), 4-7: 38.4% (133/384), 8-10: 55.8% (231/384), CS: 0-3: 2.2% (2/93), 4-7: 32.2% (29/93), 8-10: 65.6% (62/93) | | **NA** |
|  | **Legend: Abbreviations:** RCT = Randomized Controlled Trial, LIC = Low-Income Country, LMIC = Low-Middle Income Country, NICU = Neonatal Intensive Care Unit, IRR = Incidence Rate Ratio, CI = Confidence Interval, CS = Caesarean Section, OR = Odds Ratio, RR = Relative Risk, HIE = Hypoxic Ischemic Encephalopathy, EFM = Electronic Foetal Monitoring, IA = Intermittent Auscultation, FSB = Fresh Stillbirth, MSB= Macerated Stillbirth | | | | | | |  |
