# Supplementary material for: Strategies for intrapartum foetal surveillance in low- and middle-income countries: A systematic review
Source: PLoS One. 2018 Oct 26;13(10):e0206295. doi: 10.1371/journal.pone.0206295 (PMC6203373; doi:10.1371/journal.pone.0206295)
Supplement: S3 Table — (DOCX) [file pone.0206295.s008.docx]

**S3 Table. Characteristics and results of the observational studies (n=32)**

| **Study** | **Strategy of foetal surveillance** | | **Study Characteristics** | **Facility characteristics** | **Strategy of implementation** | | | **Study population** |  | | **Neonatal Outcomes** |  |  |  |  | **Test performance** |
| --- | --- | --- | --- | --- | --- | --- | --- | --- | --- | --- | --- | --- | --- | --- | --- | --- |
| **Partograph** | | | | | | | | | | | | | | | | |
| Bolbol-Haghighi et al. 2015 | Partograph. | Prospective cross-sectional study. Iran (UMIC). | | **NA** | **NA** | | N=140 women. | | | **ABNORMAL FHR: Left of the alert line**: 11.45% (11/96), **Right of the alert line**: 37.5% (15/40)  **RESUSCITATION: Left of the alert line: Need** 19.8% (19/96), **No need:** 80.2% (77/96), **Right of the alert line: Need:** 97.5% (39/40), **No need:** 2.5% (1/40), | | | | | | **Resuscitation:**  Sens: 97.5%, 100%, Spec: 80.2%, 88%, PPV: 97.3%, 62.5, NPV: 98.7%, 100  s |
| Dujardin et al, | Partograph | Prospective cohort study, Senegal (LIC). | | Four peripheral maternity clinics. | Time between alert and action lines on partograph was 3hours. | | N=1022 | | | **FRESH STILLBIRTHS: Normal labour:** 1.0% (9/919), **Only alert line crossed:** 3.1%(2/64), RR:3.2(0.7-14.5), p<0.001. **Alert line and action line crossed:** 9.7%(3/31), RR:9.9(2.8-34.7), p<0.001  **MACERATED STILLBIRTHS**: **Normal labour**: 1.3% (12/919. **Only alert line crossed:** 3.1%(2/64, RR:2.4(0.5-10.5). **Alert and action line crossed:** 0%(0/31)  **Neonatal resuscitation: Normal labour:** 4.3% (38/880), **Only alert line** **crossed:** 17.2%(10/58), RR:4.0(2.1-7.6), p<0.0001. **Alert line and action line crossed:** 17.4%(4/23), RR:4.0(1.6-10.3), p=0.02. | | | | | | **Resuscitation:**  Sens 27%, 8%  Spec: 93%, 98%  PPV: 17%, 17% |
| Ogwang et al. 2009 | Partograph. | Cross-sectional study. Uganda (LIC). | | 8 health facilities: Hospital and HC IIIs: public and private non-profit health facilities. | At each hospital at least one midwife was trained to use the partograph. | | N=1170 women. | | | **APGAR <7: FHR: Substandard partograph use:** 6.8% (29/426), **Standard:** 1.9% (14/744), **Cervical dilatation: Substandard**: 14.7% (35/238), **Standard:** 3.1% (23/735), **Action-line crossed: Yes**: 38.5% (5/13), **No:** 20.7% (199/960), **Uterine contractions: Substandard:** 9.1% (33/364), **Standard:** 5.3% (21/396) | | | | | | **NA** |
| **Meconium** | | | | | | | | | | | | | | | | |
| Duhan et al. 2010 | Meconium. | | Prospective case-control study. India (LMIC). | Tertiary care centre. | **NA** | N=1267 meconium/clear liquor  (n=100/ n=1167). | | | **STILLBIRTH: Clear:** 0% (0/1167), **LMS:** 0% (0/39)**, MMS:** 0% (0/43)**, TMS:** 50% (1/18)  **APGAR <7 AT 1 MIN: Clear:** 5.3% (63/1167), **LMS:** 5.1% (2/39)**, MMS:** 11.6% (5/43)**, TMS:** 50% (9/18)  **APGAR <7 AT 5 MIN: Clear:** 1.1% (13/1167), **LMS:** 0% (0/39), **MMS:** 0% (0/43), **TMS:** 0.2% (1/18)  **ABNORMAL FHR: CTG patterns: Normal:** Clear**:** 75.0%(875/1167), LMS: 64.1%(25/39), MMS: 65.1%(28/43), TMS: 55.6%(10/18), **Suspicious:** 14.0%(163/1167), 25.6%(10/39), 23.3%(10/43), 27.8% (5/18), **Abnormal:** 11.1% (129/1167), 10.3%(4/39), 11.6%(5/43), 16.7% (3/18), p(suspicious FHR patterns vs normal)<0.01  **CS: Total: Clear:** 17.1% (200/1167), **LMS:** 17.9% (7/39), **MMS:** 18.6% (8/43), **TMS:** 33.3% (6/18), **LSCS for foetal distress: Clear:** 3.7% (43/1167), **LMS**: 2.6% (1/39), **MMS:** 4.7% (2/43), **TMS:** 11.1% (2/18), p(TMS group vs clear)=p<0.01  **INSTRUMENTAL VAGINAL DELIVERY: Total: Clear:** 17.8% (208/1167), **LMS** 20.5% (8/39), **MMS:** 25.6% (11/43), **TMS:** 27.7% (5/18), **For foetal distress: clear:** 4.3% (50/1167), **LMS:** 2.6% (1/39), **MMS:** 4.7% (2/43), **TMS:** 11.1%(2/18). No significant difference  **NICU ADMISSION: Birth weight >2500 gm: Clear:** 2.9%(34/1176), **LMS:** 2.6% (1/39), **MMS:** 2.3% (1/43), **TMS:** 11.1% (2/18), **Birth weight <2500 gm: Clear:** 4.6% (54/1167), **LMS:** 2.6% (1/39), **MMS:** 4.56% (2/43), **TMS** 11.11% (2/18), p(TMS vs all other groups)=<0.01  **NOTE:** Clear, light -(LMS), moderate- (MMS) or thick meconium staining (TMS). | | | | | | | **NA** |
| Gupta et al, 1995 | Meconium stained amniotic fluid | | Prospective cohort, India (LMIC) | University hospital | **NA** | N=1426, MSAF/Non-MSAF (n=204/1222) | | | **FOETAL DISTRESS: MSAF:** 24.5%(50/204), **Non-MSAF:** 4.1%**(**50/1222), x^2^ value=111.72, p<0.001  **APGAR <7 AT 1 MIN: MSAF: 24.5%(50/204), Non-MSAF:** 6.3%(77/1222), p<0.001  **NEONATAL DEATH: MSAF:** 4.9%(10/204), **Non-MSAF:** 2.8%(34/1222), p value insignificant, thick MSAF: 5.7%(8/141), Non-MSAF: 0%(0/63).  **MAS: thick MSAF:** 6.4%(9/141), **thin MSAF:** 0%(0/63 | | | | | | |  |
| **Foetal Scalp Stimulation Test** | | | | | | | | | | | | | | | | |
| Rathore et al. 2011 | Foetal scalp stimulation with intermittent auscultation. | | Prospective cohort study. India (LMIC). | Urban, tertiary care teaching hospital. | **NA** | N=750 women. | | | **NEONATAL DEATH BEFORE DISCHARGE: Reactive:** 0.5%(3/581), **Non-reactive:** 5.3%(13/169)  **APGAR <5 AT 1 MIN:** **Median (range)**: **Reactive:** 9(3-9), **Non-reactive** 9(0-9), p<0.001  **APGAR <5 AT 5 MIN: Reactive** 0% (0/581), **Non-reactive** 2.3% (4/169). **Median (range)**: **Reactive:** 9(7-9), **Non-reactive** 9(0-9), p<0.001  **UMBILICAL CORD pH: pH <7.2: Reactive:** 17.4% (96/581), **Non-reactive**: 43.7% (66/169), **pH <7.0: Reactive:** 2.2% (12/581), **Non-reactive:** 12.6% (19/169)  **NICU ADMISSION: Reactive:** 0.9% (5/581), **Non-reactive:** 7.6% (13/169)  **ASPHYXIA RELATED MORBIDITY: Reactive**: 0.7% (4/581), **Non-reactive:** 5.3% (9/169)  **NOTE: Authors’ definition of reactivity:** rise of FHR by 15 bpm and the acceleration lasted more than 15 s. **Non-reactivity:** FHR failed to rise at all, if the rise was less than 15 bpm or the rise was not sustained for 15 s or if the FHR decelerated. | | | | | | | **FSST to pH <7.2**: Sens: 40.7%, Spec: 84.2%, PPV: 43.7%, NPV: 82.7%, LR+: 2.6, LR-: 0.7  **FSST to pH <7.1**: Sens: 59.4%, Spec: 81.2%, PPV: 24.3%, NPV: 95.2%, LR+: 3.2, LR-: 0.5  **FSST to pH <7.0**: Sens: 61.4%, Spec: 80.4%, PPV: 12.6%, NPV: 97.8%, LR+: 3.1, LR-: 0.5 |
| **Foetal Acoustic Stimulation Test** | | | | | | | | | | | | | | | | |
| Chittacharoen et al. 1997 | FAST: Maternal perception of sound-provoked foetal movement | | Prospective cohort study. Thailand (UMIC). | University hospital |  | N=739 | | | **POOR PERINATAL OUTCOMES: Normal(positive) response:**0% (0/653), **Abnormal (negative) response:** 15.1%(13/86)  **NOTE: Author’s definition of poor perinatal outcome**: thick meconium stained amniotic fluid, perinatal death, a 5 min Apgar score less than 7, admission to the neonatal intensive care unit or foetal distress requiring caesarean deliver | | | | | | | **Poor perinatal outcome:**  Sens 100%,  Spec 89.9%  PPV: 15.1%  NPV: 100%,  accuracy 90.1%, positive predictive value 15.1%. |
| Chittacharoen et al. 2000 | FAST and admission CTG. | | Prospective cohort study. Thailand (UMIC). | Urban. | **NA** | N=307 women. High-risk. | | | **POOR NEONATAL OUTCOME: ADMISSION TEST: abnormal test:** 45.2% (14/31), **normal test** 5.8% (16/276), **FAST: abnormal FAST**: 65.5% (19/29), **normal FAST:** 4.0% (11/278)  **NOTE: Authors’ definition of poor perinatal outcome:** presence of thick meconium stained amniotic fluid, perinatal death, a 5 min Apgar score < 7, NICU admission or foetal distress requiring operative delivery. **Foetal distress:** persistently ominous foetal heart rate pattern foetal heart rate <120 or >180 bpm. | | | | | | | **Admission Test:** Sens: 46.7%, Spec: 93.8%, PPV: 45.2%, NPV: 94.2%, DA: 89.3%  **FAST:** Sens: 63.3%, Spec: 96.4%, PPV: 65.5%, NPV: 96%, DA: 93.1% |
| **Intermittent Auscultation** | | | | | | | | | | | | | | | | |
| Adanikin en al. 2016 | Intermittent auscultation by foetoscope or Doppler. | | Retrospective cross-sectional study. Nigeria (LMIC). | Urban, government tertiary health facility. Annual deliveries: 2.500. One functioning CTG machine available, not usually used intrapartum. | Use of standard methods recommended by e.g., FIGO guidelines for monitoring FHR. | N=301 neonates. Cases suspected of intrapartum foetal distress. Low-risk: 20.3%. | | | **PERINATAL MORTALITY RATE:** 47/1000  **ABNORMAL FHR:** 82.7%, excluding meconium stained liquor and no obvious basis  **VAGINAL DELIVERY: Total:** 25.9% (78/301), **Asphyxia:** 23.1% (18/78), **No asphyxia:** 76.9% (60/78), AOR: 1.00 (0.55-1.86)  **CS:** **Total:** 74.1% (223/301), **Asphyxia:** 23.3% (52/223), **No asphyxia:** 76.7% (171/223), AOR: 1.00, p=0.965  **INTRAPARTUM FOETAL DISTRESS: Tachycardia:** 47.8% (144/301), **Bradycardia:** 34.9% (105/301), **Meconium:** 0.3% (1/301)**, Meconium + Tachycardia:** 8.3% (25/301), **Meconium + Bradycardia:** 7.3% (22/301)  **APGAR <7 AT 5 MIN: Tachycardia: Asphyxia:** 22.2% (32/144), **No asphyxia:** 77.9% (112/44) AOR:1.00, **Bradycardia:** **Asphyxia:** 27.6% (29/105), **No asphyxia:** 72.4% (76/72.4), AOR: 0.42-1.34, p=0.328, **Meconium**: **Asphyxia:** 0% (0/1), **No asphyxia**: 100% (1/1), AOR: -0.35-0.79, p=0.593, **Tachycardia + Meconium**: **Asphyxia:** 20% (5/25), **No asphyxia**: 80% (20/25), AOR: 0.41-3.22, p=0.804, **Bradycardia + Meconium**: **Asphyxia:** 18.2% (4/22), 81.8% (18/22), AOR: 0.42-3.97, p=0.668, **None: Asphyxia:** 0% (0/4), **No asphyxia**: 100% (4/4), AOR: 2.60, 0.19-36.51  **NOTE: Authors’ definition of low-risk:** women with no known pregnancy complications prior to labour onset. **High-risk:** 79.7% (others). | | | | | | | **NA** |
| Langli Ersdal et al. 2012 | Intermittent auscultation by stethoscope. | | Prospective cohort study. Tanzania (LIC). | Rural referral hospital. | **NA** | N=10271 neonates. | | | **STILLBIRTH (FRESH, MACERATED): Normal FHR:** 0.3% (30/9649), 0.1% (13/9649), **FHR not measured:** 3.5% (5/143), 4.9% (7/143) **FHR abnormal:** 10.4% (29/279), 1.1% (3/279), **FHR non-detected:** 47.5% (95/200), 48.5% (97/200), OR = 1,983 (95% CI: 922–4,264, p≤0.0005)  **NORMAL AT 24 HOURS: Normal FHR:** 98.6% (9512/9649), **FHR not measured**: 91.6% (131/143), **FHR abnormal**: 79.6% (222/279), **FHR non-detected**: 2.5% (5/200), Probability=0.08 (95% CI: 0.04–0.15)  **NICU: Normal FHR:** 0.3% (26/9649), **FHR not measured:** 0% (0/143), **FHR abnormal:** 2.5% (7/279), **FHR non-detected:** 0% (0/200)  **NEONATAL DEATH AT 24 HOURS: Normal FHR:** 0.7% (68/9649), **FHR not measured:** 0% (0/143), **FHR abnormal:** 6.5% (18/279), **FHR non-detected:** 1.5% (3/200)  **CS: Normal FHR:** 10.6% (1019/9649), **FHR not measured:** 0% (0/143), **FHR abnormal:** 76.0% (212/279), **FHR non-detected:** 20% (40/200)  **VACUUM DELIVERY: Normal FHR:** 0.7% (70/9649), **FHR not measured:** 0% (0/143), **FHR abnormal:** 3.9% (11/279), **FHR non-detected:** 3.5% (7/200)  **ABNORMAL FHR: Labour complications:** OR: 31.4 (95% CI: 23.1-42.8), **CS:** OR: 26.8 (95% CI: 20.2-35.5, p≤0.0005, **Face mask ventilation:** OR: 7.8 (95% CI: 5.9-10.1), p≤0.0005, **Apgar score <7** **at 5 min:** OR: 21.7 (95% CI: 12.7-37.0), p≤0.0005, **Neonatal death within 24hrs:** OR: 9.9 (95% CI: 5.6-17.5), p≤0.0005, **NICU:** OR: 3.0 (95% CI: 1.2–7.8), p=0.023, **Fresh stillbirth:** OR: 35.0 (95% CI: 20.3-60.4), p≤0.0005 | | | | | | | **NA** |
| **ELECTRONIC FHRM** | | | | | | | | | | | | | | | | |
| Aboulghar et al. 2013 | CTG. | | Prospective cohort study. Egypt (LMIC). | Urban. University Maternity Hospital. | **NA** | N=100 caesarean sections. High-risk. | | | **APGAR ≤4 AT 1 MIN: Pathological CTG:** 29.2% (14/48), **>4**: 70.8% (34/48). **Suspicious CTG: ≤4**: 9.6% (5/52), **>4:** 90.4% (47/52), p=0.002  **APGAR ≤6 AT 5 MIN: Pathological CTG ≤6:** 8.3% (4/48), **>6:** 91.7% (44/48). **Suspicious CTG: ≤6:** 3.8% (2/52), **>6:** 96.2% (50/52), p=0.42  **UMBILICAL CORD pH: Pathological CTG: <7.2:** 50% (24/48), **≥7.2:** 50% (24/48), **Suspicious CTG: <7.2:** 19.2% (10/52), **≥7.2:** 76.9% (42/52), p=0.002  **NICU ADMISSION: Pathological CTG: Yes:** 33.3% (16/48), **No:** 66.7% (32/42), **Suspicious CTG: Yes:** 23.1% (12/52), **No:** 76.9% (40/52), p=0.27  **ABNORMAL FHR AND pH<7.2: Normal baseline FHR:** 80% (8/10), **Normal baseline:** 46.4% (26/56), RR: 1.4 (95% CI: 0.76-2.57), not significant, **Tachycardia:** 70% (7/10), **Normal baseline:** 46.4% (26/56), RR: 1.3 (95% CI: 0.68-2.49), not significant, **Bradycardia:** (1/0), **Normal baseline:** 46.4% (26/56), RR: 3.2 (95% CI: 2.3-2.57), significant, **Non-reassuring variability:** 27.2% (3/11), **Normal variability:** 24% (6/25), RR: 1.11 (95% CI: 0.32-3.8), not significant, **Abnormal variability:** 83.3% (25/30), **Normal variability:** 24% (6/25), RR: 2.35 (95% CI: 1.08-5.09), significant, **Absent accelerations:** 54.1% (33/61), **Accelerations:** 20% (1/5), RR: 2.1 (95% CI: 0.35-12.87), not significant, **Early decelerations:** 0% (0/1), **Absent early decelerations:** 51.5% (34/66), **Late decelerations:** 150% (10/5), **Absent late decelerations:** 39.3%(24/61) RR: 7.1 (95% CI:3.86-12.3), significant, **Typical variable decelerations:** 29.4% (5/17), **Absent typical; variable decelerations:** 46.5% (20/43) RR: 0.7 (95% CI:0.31-1.68), not significant, **Atypical variable decelerations:** 150% (9/6), **Absent atypical variable decelerations:** 46.5% (20/43) RR:1.9 (95% CI:1.1-3.27), significant, **Sinusoidal pattern CTG:** 200% (2/1), **Absent sinusoidal pattern CTG:** 49.2% (32/65), RR: 0.7 (95% CI: 0.31-1.68), not significant, **Pathological:** 100% (24/24), **Suspicious:** 23.8% (10/42), RR: 2.6 (95% CI:1.39-4.85), significant  **NOTE: Authors’ definition of high-risk:** antepartum haemorrhage, pre-labour rupture of membrane, pregnancy associated with medical disorders etc. and women who delivered by CS based upon the presence of pathological or suspected pathological CTG findings suggestive of foetal hypoxia | | | | | | | **NA** |
| Bogdanovic et al. 2014 | Intermittent CTG.  Healthy newborns. | | Retrospective case-control study. Bosnia (UMIC). | Urban. Gynaecological-obstetrics clinic. | **NA** | N=108 (n=68 HIE/n=40). | | | **PATHOLOGICAL CTG: HIE:** 66.2% (45/68), **Control group:** 27.5% (11/40), p<0.05  **HIE**: **Bradycardia:** 13.2% (9/68), **Tachycardia:** 16.2% (11/68), **Silent type:** 11.8% (8/68), **Late decelerations:** 22.1% (15/68), **Variable decelerations:** 10.3% (7/68)  **APGAR SCORE AT 5 MIN <7:** **HIE:** 17.64(12/68)  **MODE OF DELIVERY: HIE: Vaginal delivery:** 79.4% (54/68)  **CS:** 17.5%(12/68), **Vacuum delivery:** 2.9% (2/68)  **NOTE: Authors’ definition of pathological CTG:** bradycardia <100, tachycardia >180, silent type of curve or late decelerations. **HIE:** based on: Apgar score at 1 and 5mins, clinical features, neurological status and ultrasound of the brain. | | | | | | | **Pathological CTG for HIE:** Sens: 66%, Spec: 27%, PPV: 80%, False +: 27.5%, False -: 33.8% |
| Odendaal et al. 1977 | Spiral scalp electrodes. | | Prospective cohort study. South-Africa (UMIC). | **NA** | **NA** | N=948 neonates. | | | **APGAR <7 AT 5 MIN: Accelerations**: 15% (48/310), **No accelerations:** 5% (30/638)  **SGA**: **Acceleration:** 20% (62/310), **No acceleration:** 13% (83/638) | | | | | | | **NA** |
| Roy et al. 2008 | CTG. | | Prospective cohort study. India (LMIC). | Tertiary care centre. | **NA** | N=217 caesarean sections. | | | **APGAR <7 AT 5 MIN: Persistent bradycardia:** 15.1% (16/106), **Recurrent late deceleration:** 17.9% (10/56), **Variable deceleration:** 18.4% (7/38), **Decreased beat to beat variability:** 0% (0/17)  **UMBILICAL CORD PH <7.10: Persistent bradycardia:** 3.8% (4/106), **Recurrent late deceleration:** 8.9% (5/56), **Variable deceleration:** 10.5% (4/38), **Decreased beat to beat variability**: 0% (0/17)  **NICU ADMISSION: Persistent bradycardia:** 15.1% (16/106), **Recurrent late deceleration:** 17.9% (10/56), **Variable deceleration:** 18.4% (7/38), **Decreased beat to beat variability**: 0% (0/17) | | | | | | | **NA** |
| Tasnim et al. 2009 | CTG. | | Prospective cross-sectional study. Pakistan (LMIC). | **NA** | **NA** | N=57 woman undergoing CS. Decision of CS was based on suspicious CTG in 51 (89%) cases and pathological CTG in 6 (11%) cases. | | | **UMBILICAL CORD: pH<7.2: Suspicious CTG:** 17.6% (9/51), **Pathological CTG:** 100% (6/6), **PCO2 >50 mmHG: Suspicious CTG**: 13% (7/51), **Pathological CTG**: 83.3% (5/6), **PO2<18 mmHG: Suspicious CTG**: 13% (7/51), **Pathological CTG**: 50% (3/6), **Base excess ≥6: Suspicious CTG**: 35% (18/51), **Pathological CTG**: 66% (4/6) | | | | | | | **PPV: pH<7.2:** CTG suspicious/pathological: 17.6%,100%  **PPV: PCO2 >50 mmHG:** CTG suspicious/pathological: 13%, 83.3%  **PPV: PO2<18 mmHG:** CTG suspicious/pathological: 13%, 50%  **PPV: Base excess ≥6:** CTG suspicious/pathological: 35%, 66% |
| **ADMISSION TESTS** | | | | | | | | | | | | | | | | |
| David et al. 2014 | Admission CTG. | | Prospective cohort study. India (LMIC). | Urban, private, non-profit medical institution. | **NA** | N=400 women. | | | **ABNORMAL FHR: Reactive:** 66.8%(267/400), **Equivocal:** 28.5%(114/400), **Ominous:** 4.8%(19/400)  **APGAR<7: Reactive:** 4.87%(13/267), **Equivocal** 10.5% (13/19), **Ominous** 68.4% (13/19), p<0.0001  **CS: Reactive:** 30.0%(80/267), **Equivocal:** 92.1%(105/114), **Ominous:** 100%(19/19), P < 0.05, **Foetal distress as indication for CS: Reactive:** 3.75% (3/80), **Equivocal:** 12.4% (0513/), **Ominous:** 47.4% (9/819), p<0.001  **VAGINAL: Reactive:** 67.0% (179/267), **Equivocal:** 1.8% (2/114), **Ominous:** 0% (0/19), p<0.0001  **FORCEPS: Reactive:** 3% (8/267), **Equivocal:** 6.1% (7/114), **Ominous:** 0% (0/19), p<0.0001  **MECONIUM: Moderate-thick: Reactive:** 9% (24/267), **Equivocal:** 39% (44/114), **Ominous:** 72% (14/19), p<0.001  **NICUADMISSION: Reactive:** 1.1% (3/267), **Equivocal:** 12.3% (14/114), **Ominous:** 47.4% (9/19), p=0.03 | | | | | | | **Foetal outcome:** Sens: 92.85%, Spec: 94.2%, PPV: 88.0%, NPV: 96.6%, DA: 93.8% |
| Goldenberg et al. 2013 | Admission IA (doptone or other device). | | Prospective cross sectional study. Pakistan, India, Kenya, and Democratic Republic of Congo (DRC) (LIC and LMIC). | Urban (Pakistan), semi-urban (India) and rural (Kenya). All annual deliveries >1000, mid-level public hospitals, caesarean delivery and neonatal care available (except Pakistan). | Each hospital was given 2 doptones and the staff, including physicians, midwives, and nurses, were given a single demonstration regarding their use by a project supervisor. | N=3593 neonates, n=3555 woman. Woman in labour admitted to the labour ward. | | | **STILLBIRTH (MACERATED, FRESH): Total:** 0.92% (33/3593), 1.48% (53/3593), **FHT present:** 0.09% (3/3448), 0.46% (16/3448), **FHT absent:** 40% (26/65), 49.23% (32/65), **Unknown FHT:** 5% (4/80), 6.25% (5/80), **≥2500g: Total**: 0.56% (17/3050), 1.05% (32/3050), **FHT present:** 0.03% (1/2969), 0.44% (13/2969), **FHT absent:** 38.9% (14/36), 50% (18/36), **Unknown FHT:** 0.44% (2/45), 0.22% (1/45)  **ALIVE AT DISCHARGE: Total:** 96.66% (3472/3593). **FHT present:** 98.69% (3403/3448), **FHT absent:** 6.15% (4/65), **Unknown FHT:** 81.25% (65/80), **≥2500g: Total:** 97.80% (2983/3050), **FHT present:** 99.1% (2941/2969), **FHT absent:** 5.56% (2/36), **Unknown FHT:** 88.89% (40/45)  **NEONATAL DEATH BEFORE DISCHARGE: Total:** 0.97% (35/3593). **FHT present:** 7.47% (26/3448), **FHT Absent:** 4.62%(3/65), **Unknown FHT:** 7.5% (6/80), **≥2500, Total:** 0.60% (18/3050), **FHT present:** 0.47% (14/2969), **FHT absent:** 0.56% (2/36), **FHT unknown:** 0.44% (2/45)  **PERINATAL DEATH: Total:** 3.37% (121/3593), **Rate:** 34/1000 deliveries, **FHT present:** 1.31% (45/3448), **Rate:** 13/1000 deliveries, **FHT absent:** 93.85% (61/65), **Rate:** 938/1000 deliveries, **Unknown FHT:** 18.75% (15/80), **Rate:** 187/1000 deliveries, **≥2500, Total:** 2.2% (67/3050), **Rate:** 22/1000 deliveries, **FHT present:** 0.94% (28/2969), **Rate:** 94/1000 deliveries, **FHT absent:** 94.44% (34/36), **Rate:** 944/1000 deliveries, **Unknown FHT:** 11.1% (5/45), **Rate:** 111/1000 deliveries.  **In-hospital deaths:** 1.3% (45/3448), 40% of all perinatal mortality  **NOTE: Authors’ definition of Perinatal mortality:** all stillbirths or neonatal deaths before hospital discharge. | | | | | | | **NA** |
| Rahman et al. 2012 | Admission CTG. | | Prospective cohort study. India (LMIC). | Teaching hospital. | **NA** | N=176 women. | | | **NICU ADMISSION: Reactive:** 3.5% (5/145), **Equivocal:** 27.8% (5/18), **Ominous:** 61.5% (8/13)  **APGAR <7 AT 5 MIN: Reactive:** 3.5% (5/145), **Equivocal:** 27.8% (5/18), **Ominous:** 61.5% (8/13)  **ABNORMAL FHR: Reactive**: 82.3% (145/176), FD: 6.9% (10/145), **Equivocal**: 10.2% (18/176), FD: 39.9% (7/18), **Ominous**: 7.4% (13/176), FD: 84.6% (11/13), p<0.001  **CS: Reactive**: Total: 20.7% (30/145), FD: 20% (6/30), **Equivocal**: Total: 44.5% (8/18), FD: 50% (4/8), **Ominous:** Total: 76.9% (10/13), FD: 100% (10/10), p<0.001  **Spontaneous DELIVERY: Reactive:** Total: 70.34% (102/145), FD: 2.0% (2/102), **Equivocal:** Total: 50% (9/18), FD: 22.2% (2/9), **Ominous:** Total: 15.4% (2/13), FD: 0% (0/2) p<0.001  **FORCEPS/VENTOUSE: Reactive:** Total: 9.0% (13/145), FD: 15.4% (2/13), Equivocal: Total: 5.5% (1/18), FD: 100% (1/1**), Ominous:** Total: 7.7% (1/13), FD: 100% (1/1)  **UMBILICAL CORD pH <7.2: Reactive:** 3.5% (5/145), **Equivocal:** 16.7% (3/18), **Ominous:** 53.9% (7/13)  **MECONIUM: Reactive:** 4.8% (7/145), **Equivocal:** 33.3% (6/18), **Ominous:** 61.5% (8/13)  **NEONATAL DEATH: Reactive:** 0% (0/145), **Equivocal:** 5.6% (1/18), **Ominous:** 7.7% (1/13)  **NOTE: Authors’ definition of foetal distress:** any of the following: ominous FHR changes led to LSCS/forceps/ventouse delivery, presence of moderate-thick meconium stained liquor, Apgar score at 5 minutes < 7, umbilical cord arterial blood pH <7.2, NICU admission for birth asphyxia, neonatal seizures within first 24 hrs to 48 hrs, incidence of intrapartum/neonatal mortality | | | | | | | **Foetal distress:** Sens: 60%, Spec: 94.8%, PPV: 56.8%, NPV: 88.6%, False +: 40%, False -: 13.9% |
| Kushtagi et al. 2011 | Admission amniotic fluid index and admission CTG. | | Prospective cohort study. India (LMIC). | **NA** | **NA** | N=326 women. High and low-risk (defined by Minnesota scoring system). | | | **APGAR <7 AT 5 OR 1 MIN: <5 cm**: 8.3% (1/12), **5.1-8 cm**: 0.9% (1/116), **8.1-20 cm:** 0% (0/198)  **ABNORMAL FHR: Up to 5 cm: Reactive LAT +** Non-reassuring**:** 50% (1/2), Reassuring: 50% (1/2), **Nonreactive LAT +** Non-reassuring**:** 80% (8/10), Reassuring: 20% (2/10), **5.1-8 cm: Reactive LAT +** Non-reassuring**:** 21.9% (21/96), Reassuring: 78.1% (75/96), **Nonreactive LAT +** Non-reassuring**:** 55% (11/20), Reassuring**:** 45% (9/20), ≥**8 cm + Reactive LAT** + Non-reassuring**:** 5.4% (10/187), Reassuring**:** 94.6% (177/187), **Nonreactive LAT** **+** Non-reassuring: 18.2% (2/11), Reassuring: 81.8% (9/11)  **CS: <5 cm:** 83.3% (10/12), **5.1-8 cm:** 20.7% (24/116), **8.1-20 cm:** 6.1% (12/198)  **INSTRUMENTAL VAGINAL DELIVERY: <5 cm:** 16.7% (2/12), **5.1-8 cm:** 10.3% (12/116), **8.1-20 cm:** 2.0% (4/198)  **MAS: <5 cm:** 66.7% (8/12), **5.1-8 cm:** 2.6% (3/116), **8.1-20 cm:** 1.5% (3/198)  **UMBILICAL CORD BLOOD pH <7.1: <5 cm:** 8.3% (1/12), **5.1-8 cm:** 0% (0/116), **8.1-20 cm:** 0% (0/198)  **NICU ADMISSION: <5 cm:** 75% (9/12), **5.1-8 cm:** 19.8% (23/116), **8.1-20 cm:** 7.0% (14/198)  **NOTE: Authors’ definition of non-reassuring foetal status:** any of the following: meconium, foetal heart trace abnormality, Apgar scores <7 at 1 and 5 min, NMI of 46 or NICU admission | | | | | | | **Foetal compromise: aAFI**: Sens: 58%, Spec: 64%, PPV: 24%, NPV: 88%  **Foetal compromise: LAT**: Sens: 40%, Spec: 92%, PPV: 51%, NPV: 89% |
| Kulkarni et al. 1998 | Admission CTG. | | Prospective cohort study. India (LMIC). | CS and operative vaginal deliveries available. | **NA** | N=100. High-risk. | | | **APGAR <7 AT 5 MIN: Reactive:** 3.4% (2/58), **Equivocal:** 8.6% (3/35), **Ominous:** 28.6% (2/7) **Total:** 11% (11/100)  **FOETAL DISTRESS: Reactive:** 6.9% (4/58), **Equivocal:** 31.4% (11/21), **Ominous:** 85.7% (6/7), **Total:** 21% (21/100) **FD:** **Decelerations:** Late: 60% (9/15), Variable: 30.8% (4/13), Early: 17.6% (3/17), None: 9.1% (5/55), p<0.05, **Depth of decel.** **<60: FD:** <60: 22.2% (8/36), ≥60: 88.9% (8/9), p<0.01, **Variability:** <10, **FD:** 26.9% (17/63), ≥10: 10.8% (4/37), **FD:** 10.8% (4/37), p=0.1  **CS + OPERATIVE VAGINAL: Foetal distress: Reactive** 5.2% (3/58), **Equivocal** 22.8% (8/35), **Ominous:** 28.5% (2/7), **Other reasons: Reactive** 24.1% (14/58), **Equivoca**l 11.4% (4/35), **Ominous:** 0% (0/7)  **VAGINAL DELIVERY: Reactive:** 70.7% (41/58), **Equivocal:** 65.7% (23/35), **Ominous:** 71.4% (5/7)  **MECONIUM: Reactive:** 0% (0/58), **Equivocal:** 14.3% (5/35), **Ominous:** 85.7% (6/7)  **NOTE: Authors’ definition of perinatal morbidity:** indicated by the FHR abnormality requiring operative delivery and/or passage of thick meconium and/or Apgar score < 7 at 5 mins. **Foetal distress:** not defined. **High-risk**: pregnancies with PIH, post-datism (> 42 weeks), IUGR, oligohydramnios, reduced foetal movements or those with medical disorders like diabetes or chronic hypertension. | | | | | | | **NA** |
| Shaktivardhan et al. 2008 | Admission CTG. | | Prospective cohort study. India (LMIC). | **NA** | **NA** | N=150 women. High-risk. | | | **NEONATAL DEATH BEFORE DISCHARGE: Intrapartum/neonatal mortality: Normal:** 0% (0/101), **Equivocal:** 0% (0/34), **Abnormal:** 6.6% (1/15)  **APGAR AT 5 MIN: Between 4-5: Normal:** 3% (3/101), **Equivocal:** 17% (6/34), **Abnormal:** 40% (6/15), **≤3**: **Normal:** 1% (1/101), **Equivocal:** 9% (3/34), **Abnormal:** 13% (2/15)  **NICU ADMISSION: Normal:** 1% (1/101), **Equivocal:** 12% (4/34), **Abnormal:** 33% (5/15)  **FOETAL DISTRESS: Normal:** 15%(15/101), **Equivocal:** 55% (19/34), **Abnormal:** 73% (11/35)  **NOTE: Authors’ definition of high-risk:** history of recurrent pregnancy losses/previous stillbirth, pregnancy with concurrent medical illness (hypertension, Diabetes Mellitus, SLE, thrombophilias, antiphospholipid syndrome, renal disease), preeclampsia, PROM > 6 hrs Intrauterine growth restriction, oligohydramnios, postdatism, diminished foetal movements. | | | | | | | **Foetal distress:** Sens: 42.3%, Spec: 95.6%, PPV: 73.3%  A**pgar score<=5 at 5mins**: Sens: 66.7%, Spec: 93.3%, PPV: 53.3%  **NICU admission:** Sens: 83.3%, Spec: 90.9%,PPV: 33.3% |
| Raouf et al. 2015 | Non-stress test. | | Case-control study. Iran (UMIC). | Urban. Teaching Hospital. | **NA** | N=450, reactive/non reactive test results (n=150/n=300). | | | **STILLBIRTH: Study:** 2.7% (4/150), **Control:** 0% (0/300), p<0.001  **APGAR: 4-6: Study:** 1.3% (2/150), **Control:** 0.3% (1/300), p<0.001, **0-3: Study:** 2.7% (4/150), **Control:** 0% (0/300)  **ABNORMAL FHR: Bradycardia: Study:** 28% (42/150), **Control:** 3.3% (10/300), p<0.001  **CS: Study:** 42.7% (64/150), **Control:** 17.0% (51/300)  **DELIVERY MODE: Vaginal: Study:** 57.3% (86/150), **Control:** 83% (249/300), **Decent arrest: Study:** 2.7% (4/150)**, Control:** 4.7% (14/300), p=0.44  **MECONIUM: Study:** 11.3% (17/150), **Control:** 9.7% (29/300), p=0.62  **NICU ADMISSION: Study:** 16% (24/150), **Control:** 2.7% (8/300)  **ABNORMAL NEONATAL OUTCOME:** **Study:** Normal/moderate/low weight: 66.7% (100/150), 32.7% (49/150), 0.7% (1/150). **Control:** 88.3% (265/300), 11.3% (34/300), 0.3% (1/300), p= significant (not provided) | | | | | | | **NA** |
| **Umbilical Artery Doppler Velocimetry** | | | | | | | | | | | | | | | | |
| Howarth et al. 1992 | Umbilical artery Doppler velocimetry. | | Prospective cohort study. South-Africa (UMIC). | **NA** | **NA** | N=100 women. | | | **CS: No foetal compromise:** 17% (12/70), **Foetal compromise**: 40% (12/30)  **ASSISTED DELIVERY: No foetal compromise:** 9% (5/70), **Foetal compromise**: 44% (8/30)  **MECONIUM: No foetal compromise:** 13% (9/70), **Foetal compromise**: 40% (12/30)  **NEONATAL OUTCOME:** pulsability index  **NOTE: Authors’ definition of Foetal compromise:** abnormal first- or second-stage foetal heart rate traces, a 5 min Apgar score <7, or the development of HIE | | | | | | | **Neonatal outcome:** Sens: 13%, Spec: 89%, PPV: 25%, NPV: 70% |
| Stuart et al. 1993 | Doppler velocimetry. | | Prospective cohort study. South-Africa (UMIC). | Urban. | **NA** | N=36 women. High-risk. | | | **PORCELOT RATIO** | | | | | | | **NA** |
| **Multiple strategies** | | | | | | | | | | | | | | | | |
| Bakr et al. 2005 | Foetal pulse oximetry and foetal scalp pH. | | Prospective cross-sectional study. Egypt (LMIC). | University maternity hospital. Annual deliveries: 10.000. CTG available, Caesarean sections available. | **NA** | N=150 women. | | | **UMBILICAL CORD PH: Scalp pH≤ 7.2:** 71.7% (43/60), **pH>7.2:** 28.3% (17/60), **Pulse <30%:** 75% (45/60), **Pulse >30%:** 25% (15/60), **Pulse <40%:** 60% (36/60), **Pulse >40%:** 40% (24/60), p=non-significant  **ABNORMAL NEONATAL OUTCOME: pH≤ 7.2:** 81.6% (31/38), **pH> 7.2:** 18.4% (7/38), **Pulse <30%:** 89.5% (34/38), **Pulse >30%:** 10.5% (4/38), **Pulse <40%:** 76.3% (29/38), **Pulse >40%:** 23.7% (9/38), p=non-significant  **NOTE: Authors’ definition of abnormal neonatal outcome:** any of the following: 5 min Apgar score <7, secondary respiratory distress, NICU admission, neonatal arterial blood pH<7.15, or neonatal death | | | | | | | **Foetal blood pH < 7.20 (umbilical art<7.15)**: Sens: 72 (58–82) , Spec: 53 (42–63), PPV: 57 (48–65), NPV: 43 (35–51), **(abnormal neonatal outcome)**: Sens: 82 (65–91), Spec: 52 (42–61), PPV: 57 (48–64), NPV: 43 (35–51),  **Foetal ox </30**: **(umb art<7.15)**: Sens: 75 (62–85), Spec: 49 (38–60), PPV: 61 (52–68), NPV: 39 (32–48), **(abnormal neonatal outcome):** 89 (74–97), Spec: 49 (40–59), PPV: 61 (52–68), NPV: 39 (32–48),  **Foetal ox <40: (umb art)**: 60 (47–72), Spec: 31 (22–42), PPV: 65 (57–73), , NPV: 35 (27–43), **(abnormal neonatal outcome)**: 76 (59–88), Spec: 38 (30–48), PPV: 65 (57–73), NPV: 35 (27–43) |
| Goonewardene et al. 2011 | Foetal acoustic stimulation test and non-stress test | | Prospective cohort study. Sri Lanka (LMIC). | Urban, 2 hospitals (Teaching Hospital/Hospital for Women). | **NA** | N=486 women. | | | **APGAR <7 AT 5 MIN: FAST ALONE:** No movements: 45.2% (33/73), Movements+: 1.45% (6/413). **NST ALONE:** Nonreactive: 19.2% (39/203), Reactive: 0% (0/283), **NST after FAST:** Nonreactive: 73.6% (39/53), Reactive: 0% (0/433)  **ABNORMAL FHR: NST alone:** No movement + non-reactive: 25.1% (51/203), No movement + reactive: 7.8% (22/283), Movements + nonreactive: 74.9% (152/203), Movement + reactive 92.2% (261/283), **NST after FAST:** No movement + non-reactive: 81.1% (43/53), No movement + reactive: 6.9% (30/433), Movement + non-reactive: 18.9% (10/53), Movement + reactive: 93.1% (403/433)  **CS: Nonreactive NST after FAST:** 83.7% (36/43)  **MECONIUM: Nonreactive NST after FAST:** 100%  **NOTE:** 85% (413/486) of the women felt foetal movements after the FAST, and 31% (150/486) of the non-reactive NST became reactive after the FAST was applied. | | | | | | | **APGAR at 5 min: FAST:** Sens: 84.6%, Spec: 91.1%, PPV: 45.2%, NPV: 98.5%, DA: 90.5%  **NST before FAST**: Sens: 100%, Spec: 63.3%, PPV: 19.2%, NPV: 100%, DA: 66.3%  **NST after FAST:** Sens: 100%, Spec: 96.9%, PPV: 73.6%, NPV: 100%, DA: 97.1% |
| Odendaal et al. 1994 | FHR by ultrasound transducer or scalp electrode. | | Prospective cohort study. South-Africa (UMIC). | **NA** | **NA** | N=459 women. High-risk. | | | **APGAR <7 AT 5 MIN: Reactive pattern:** 3.5% (11/312), **Good variability:** 6.0% (8/134), **Poor variability:** 23% (3/13), p(reactive vs poor variability)<0.005  **CS: Reactive pattern:** 19.2% (60/312), **Good variability:** 34.3% (46/134), **Poor variability:** 76.9% (10/13), p(reactive vs good)<0.001, p(good vs poor) <0.005  **UMBILICAL CORD BLOOD: pH <7.2: Reactive pattern:** 13.1% (41/312), **Good variability:** 19.4% (26/134), **Poor variability:** 46.2% (6/13), p(reactive vs poor)<0.001, p(good vs poor)<0.005, **pH<7.1: Reactive pattern:** 1.9% (6/312), **Good variability:** 3.7% (5/134), **Poor variability:** 15.3% (2/13), p=non-significant, **BE <12%: Reactive pattern:** 2.2% (7/312), **Good variability:** 1.5% (2/134), **Poor variability:** 7.7% (1/13), p=non-significant  **SGA: Reactive pattern:** 8.8% (27/312), **Good variability:** 15.6% (21/134), **Poor variability:** 80% (10/13), p(reactive vs good)<0.05, p(good vs poor)<0.001, p(reactive vs poor)<0.001  **NOTE: Authors’ definition of Nonreactive pattern with good long-term variability:** fluctuation of 5 or more beats/min. **Nonreactive pattern** **with poor long-term variability:** less than 5 beats/min. | | | | | | | **NA** |
| Odongo et al. 2010 | CTG and meconium stained liquor. | | Prospective cohort study. Kenya (LMIC). | Urban, tertiary care facility, teaching hospital for post graduate medical students and advanced nursing programmes. | **NA** | N=77 (n=36/n=41). | | | **APGAR <7 AT 1 MIN: <7 + Clear:** 9.8% (4/41), **Meconium:** 25% (9/36) RR: 0.39 (CI 0.13-1.16), **CTG Normal rate:** 16.9% (12/71), **CTG Abnormal rate:** 16.7% (1/6), **CTG Normal decelerations**: 10% (2/20), **CTG Abnormal decelerations:** 19.3% (11/57), **≥ 7 + Clear:** 90.2% (37/41), **meconium:** 75% (27/36), **CTG normal rate**: 83.1% (59/71), RR: 1.01 (CI 0.16-6.53), **CTG Abnormal rate**: 83.3% (5/6), RR: 1.00 (CI 0.69-1.45), **CTG Normal decelerations:** 90% (18/20), RR: 0.13 (CI 0.12-1.08), **CTG Abnormal decelerations:** 80.7% (46/57), RR: 0.09 (CI 0.09-1.083)  **CS: Clear:** 17.1% (7/41), **Meconium**: 38.9% (14/36), RR: 1.36 (CI 1.01-1.82)  **SVD: Clear:** 82.9% (34/41), **Meconium:** 61.1% (22/36), RR: 0.44 (CI: 1.49 (CI 0.93-2.07), **Clear: Normal:** 19.5% (8/41), **Suspicious:** 14.6% (6/41), **Pathologic:** 65.9% (27/41), **Intrapartum CTG + Meconium: Normal:** 27.8% (10/36), **Pathologic:** 72.2% (26/36), **Clear liquor: Normal:** 24.4% (10/41), RR: 0.92 (CI 0.56-1.51), **Pathologic:** 75.6% (31/41), RR: 1.10 (CI 0.65-1.85) | | | | | | | **NA** |
| Oladapo et al. 2009 | IA by Pinard stethoscope and meconium stained liquor. | | Cross-sectional study. Nigeria (LMIC). | University teaching hospital. No access to electronic FHR monitoring, foetal blood gas or pH analysis during the period. | **NA** | N=246 CS based on foetal distress. | | | **ABNORMAL FHR: Severely compromised:** 61.7% (74/120), **Suboptimal condition:** 62.9% (73/116)  **MECONIUM: Severely compromised:** 35.0% (42/120), **Suboptimal condition:** 53.4% (62/116), **Abnormal FHR + Meconium: Severely compromised:** 38.3% (46/120) **Suboptimal condition:** 22.4% (26/116)  **CS: Total:** 5.7% (246/4335), **Foetal distress:** 22.8% (246/1077)  **APGAR SCORE AT 1 MIN: ≤3:** 48.8% (120/246), **4-7:** 47.2% (116/246)  **NOTE: Authors’ definition of severely compromised neonate:** Apgar score 0–3. **Suboptimal condition:** Apgar score of 4-7 at 1 min. **Foetal distress:** detection of abnormal FHR and/or rhythm (persistent tachycardia: >160 beat per minute or bradycardia: <110 bpm) by IA with the Pinard foetal stethoscope and/or presence of meconium in the amniotic fluid. | | | | | | | **NA** |
| Parveen et al. 2010 | CTG and meconium stained liquor. | | Prospective cross-sectional study. Pakistan (LMIC). | Tertiary care, private hospital. | **NA** | N=122 women. | | | **ABNORMAL FHR: CTG normal: Normal base excess:** 77% (94/122), **Intermediary CTG:** 5.7% (7/122), **Abnormal:** 6.5% (8/122), **Moderately acidotic: Normal** 7.3% (9/122), **Intermediary:** 0.8% (1/122), **Abnormal:** 0% (0/122), **Severely acidotic: Normal**: 1.6% (2/122), **Intermediary** 0.8% (1/122), **Abnormal** 0% (0/122)  **UMBILICAL CORD BASE EXCESS: Clear MSAF:** **Base excess normal:** 68.8% (84/122), **Thin/moderate:** 16.3% (20/122), **Thick:** 1.6% (2/122), **Moderately acidotic:** clear 8.1% (10/122), **Thin/moderate:** 2% (3/122), **thick:** 0% (0/122), **Severely acidotic:** clear 2% (3/122), **Thin/moderate:** 0% (0/122), **Thick:** 0% (0/122) | | | | | | | **Neonatal acidemia: CTG:** Sens: 15.4%, Spec: 86%, PPV: 11.6%, NPV: 89%  **MSAF:** Sens: 18.8%, Spec: 79.2%, PPV: 12%, NPV: 86% |
| Tongprasert et al. 2006 | Rapid biophysical profile (amniotic fluid index and sound-provoked foetal movement). | | Prospective cross-sectional study. Thailand (UMIC). | **NA** | **NA** | N=330. | | | **UNFAVOURABLE NEONATAL OUTCOME: AFI <5:** 5.71% (2/35), **AFI >5:** 1.35% (4/295), **SPFM yes:** 12.5% (2/16), **SPFM no:** 1.27% (4/314), **rBPP +:** 50.0% (3/6), **rBPP equivocal:** 0% (0/37), **rBPP -:** 1.0% (3/287)  **NOTE: Authors’ definition of unfavourable neonatal outcome:** foetal distress (late decelerations uncorrectable by conventional intra-uterine resuscitation or prolonged bradycardia with decreased variability), Apgar score at 5 min < 7, NICU admission and perinatal death | | | | | | | **Neonatal outcome: AFI**: Sens: 33.3%, Spec: 89.8%, PPV: 5.7%, NPV: 98.6%, DA: 88.8%  **SPFM**: Sens: 33.3%, Spec: 95.7%, PPV: 12.5%, NPV: 98.7%, DA: 94.5%  **rBPP**: Sens: 50.0%, Spec: 99.1%, PPV: 50.0%, NPV: 99.1%, DA: 98.2% |
| Rotich et al. 2006 | Intermittent auscultation and meconium stained liquor. | | Prospective cohort study. Kenya (LMIC). | Access to caesarean section and intensive care unit laboratory. | **NA** | N=116 (n=58 neonates with foetal distress/ n=58 neonates without distress) | | | **NEONATAL DEATH BEFORE DISCHARGE: Foetal distress:** 5% (3/58), **No foetal distress:** 0% (0/58)  **APGAR AT 1 MIN <7: Foetal distress:** 59.0% (34/58), **No foetal distress:** 31.0% (18/58). RR: 1.9 (1.2-2.9), p=0.003  **APGAR AT 5 MIN <7: Foetal distress:** 24.3% (14/58), **No foetal distress:** 3.4% (2/58), RR: 7.0 (CI 1.7-29.4), p=0.00)  **UMBILICAL CORD BLOOD pH: Foetal distress: <7.1:** 29% (17/58), **7.1-7.2:** 41% (24/58), **>7.2:** 29% (17/58), **No Foetal distress: <7.1**: 3% (2/58), **7.1-7.2:** 14% (8/58), **>7.2:** 83% (48/58)  **NICU ADMISSION: Foetal distress: Admitted:** 31% (18/58), **No foetal distress:** 17% (10/58)  **ALIVE AT DISCHARGE: Foetal distress:** 64% (37/58), **No foetal distress:** 83% (48/58)  **NOTE: Authors’ definition of foetal distress:** abnormal foetal heart rate and rhythm by IA together with meconium | | | | | | | **NA** |
|  | **Legend: Abbreviations:** RCT = randomized controlled trial, LIC = Low-Income Country, LMIC = Low-Middle Income Country, UMIC = Upper-Middle Income country, NICU = Neonatal Intensive Care Unit, IRR= Incidence Rate Ratio, CI = Confidence Interval, CS = Caesarean Section, OR = Odds Ratio, AOR= Adjusted Odds Ratio, RR = Relative Risk, HIE= Hypoxic Ischemic Encephalopathy, FHT= Foetal Heart Trace, MSAF=Meconium stained amniotic fluid, MAS= Meconium Aspiration Syndrome, EFM= Electronic Foetal Monitoring, IA = Intermittent Auscultation, FSB= Fresh Stillbirth, MSB= Macerated Stillbirth | | | | | | | | | | | | | | |  |
